# The longitudinal and concurrent relationship between caregiver sensitivity and preschool attachment: A systematic review and meta-analysis

**DOI:** 10.1371/journal.pone.0245061

**Published:** 2021-01-22

**Authors:** Monica C. O’Neill, Shaylea Badovinac, Rebecca Pillai Riddell, Jean-François Bureau, Carla Rumeo, Stefano Costa

**Affiliations:** 1 Department of Psychology, York University, Toronto, Ontario, Canada; 2 Department of Psychiatry Research, Hospital for Sick Children, Toronto, Ontario, Canada; 3 Department of Psychiatry and Pharmacy, University of Toronto, Toronto, Ontario, Canada; 4 School of Psychology, University of Ottawa, Ottawa, Ontario, Canada; Universidade Federal dos Vales do Jequitinhonha e Mucuri, BRAZIL

## Abstract

The present study aimed to systematically review and meta-analyze the concurrent and longitudinal relationship between caregiver sensitivity and preschool attachment measured using the Main and Cassidy (1988) and Cassidy and Marvin (1992) attachment classification systems. This review was pre-registered with the International Prospective Register of Systematic Reviews (PROSPERO; Registration Number CRD42017073417) and completed according to the Preferred Reporting Items for Systematic Reviews and Meta-Analyses (PRISMA) guidelines. The present review identified 36 studies made up of 21 samples (*N* = 3, 847) examining the relationship between caregiver sensitivity and preschool attachment. Eight primary meta-analyses were conducted separately according to the proximity of the assessment of sensitivity to attachment (i.e., concurrent versus longitudinal), operationalization of caregiver sensitivity (i.e., unidimensional versus multidimensional) and attachment categorizations (i.e., secure-insecure versus organized-disorganized). Overall, the meta-analyses revealed higher levels of caregiver sensitivity among caregivers with secure and organized preschoolers, relative to insecure and disorganized preschoolers, respectively. Medium effect sizes (*g* = .46 to .59) were found for both longitudinal and concurrent associations between caregiver sensitivity and preschool attachment when a unidimensional measure of caregiver sensitivity was employed, compared to small to medium effect sizes (*g* = .34 to .49) when a multidimensional measure of caregiver sensitivity was employed. Child age at attachment measurement was a significant moderator of the longitudinal association between unidimensional caregiver sensitivity and preschool attachment. Future directions for the literature and clinical implications are discussed.

## Introduction

### The relationship between preschool attachment and mental health

Mental health disorders occur in approximately 10–20% of children and adolescents across the globe, with 50% of mental health difficulties beginning by early adolescence and 75% occurring by early adulthood [1]. Early prevention and treatment is imperative for improving developmental psychopathology across the lifespan. In order to develop programs targeting early mental health prevention, it is essential to identify and understand potential risk factors of child mental health. Early maladaptive attachment to the primary caregiver is one risk factor that has been linked with psychological disorders in childhood [2–6]. For example, insecure attachments are associated with internalizing symptomatology, while insecure and disorganized attachments are associated with externalizing symptomatology [3]. However, in order to improve child attachment and accordingly mental health, it is necessary to elucidate how and why these attachment difficulties may develop. A caregivers’ sensitivity toward their child is one factor that has been proposed as a potential predictor of child attachment [7]. This is supported by more recent reviews [5, 6], which identify that parent interventions, mostly aimed at improving parental sensitivity, are related to decreased disorganized attachment outcomes. A review of the literature investigating the intricate relationship between caregiver sensitivity and preschool attachment is necessary to work toward understanding potential mechanisms of improving attachment issues and mental health from childhood through adulthood.

### Preschool attachment

Bowlby [8] postulated that early experiences with attachment figures shape children’s internal working model of the world. With repeated exposure to a sensitive and responsive attachment figure, children learn to explore the world with confidence and obtain support when necessary, thereby developing working models of a secure self, a caring attachment figure, and the world as nonthreatening [9]. Alternatively, with repeated exposure to an insensitive caregiver, children see the world as unreliable, and unpredictable.

The Ainsworth, Blehar, Waters, and Wall [10] system of attachment established the classification of infants’ attachment to their primary caregiver using the lab-based separation-reunion procedure. Although several measures have been developed to assess attachment in preschool and early childhood, the dominant approach most akin to Ainsworth and colleagues’ [10] system is that which assumes stability in attachment from infancy through to childhood [11]. In accordance with this theoretical approach, Main and Cassidy [12] developed the classification system for 6-year-olds, whereas Cassidy, Marvin, and the MacArthur Working Group [13] modified this system for preschoolers (2.5- to 4.5-years-old).

The Cassidy and Marvin [13] and Main and Cassidy [12] coding systems specify that preschoolers and young children may be classified according to one of six attachment classification patterns including; secure, avoidant, ambivalent, disorganized and/or insecure-other, controlling-caregiving, and controlling-punitive [12, 13]. Children with a secure attachment pattern demonstrate a calm and comfortable enjoyment with the caregiver, using the caregiver as a secure base to explore their environment [13]. Children with an avoidant attachment pattern demonstrate an attempt to maintain neutrality by avoiding physical and emotional interactions that may bring attention to the child-caregiver relationship [13]. Children classified with an ambivalent attachment pattern may emphasize dependency on the caregiver through immature behaviours (e.g., “baby talk”), or they may demonstrate resistant behaviours through moderate anger, resistance, or avoidance [13]. Children classified with a disorganized and/or insecure-other attachment pattern may demonstrate disordered temporal sequences, incomplete movements, confusion and apprehension, disoriented expressions, or depressed affect [13]. Children classified with a controlling-caregiving attachment pattern may demonstrate a desire to guide, orient, or cheer-up the parent, whereas children with a controlling-punitive attachment pattern may demonstrate punitive or hostile behaviours toward the parent [13].

Owed to the decades of literature resulting from the development of this system, and only one other existing related review ([14]; focusing solely on maternal depression and preschool attachment) synthesizing these systems, the present review focused exclusively on studies employing the Main and Cassidy [12] and Cassidy and Marvin [13] coding systems. Investigating the relationship between multiple aspects of maternal behaviour and infant attachment, Ainsworth and colleagues [10] first identified maternal sensitivity as the *most important* predictor of infant attachment. Given the available literature almost exclusively explored maternal sensitivity, rather than paternal sensitivity, the current review primarily focused on maternal sensitivity. However, we note a recent surge of studies on father-child attachment [15–17], suggesting that reviews focusing more on paternal sensitivity may be warranted in the future.

### Operationalizing maternal sensitivity

A construct that has been identified as integral to the development of secure attachment is maternal sensitivity [18, 19]. Key tenets of maternal sensitivity include attunement to the infant’s signals, correct interpretation of the infant’s perspective and communicated needs, and prompt and appropriate responding [18]. Since the development of Ainsworth and colleagues’ [18] original sensitivity scale and other Maternal Care scales, additional measures have been developed to assess caregivers’ sensitivity toward their infants and young children [20].

In a recent systematic review of behavioural measures developed to assess caregiver sensitivity, Mesman and Emmen [20] completed an in-depth analysis of eight instruments aimed at assessing caregiver sensitivity in comparison to Ainsworth et al.’s [18] original construct. Among the eight measures examined, only three employed a single global rating of sensitivity similar to Ainsworth et al.’s [18] original sensitivity scale [20]. In contrast to Ainsworth et al.’s [18] sensitivity scale which involves a global judgement of sensitivity, the remaining five measures required the summation of several scales to create a combined score representing sensitivity and other related behaviours (e.g., warmth, positive affect). Mesman and Emmen [20] propose that one way to advance our comprehension of the intricacies of maternal sensitivity as a construct, is to examine the contribution of a single global assessment of sensitivity in comparison to a composite assessment of sensitivity and related constructs. Accordingly, the primary focus of the present review was to examine the relationship between sensitivity and preschool attachment, according to whether the reviewed studies implemented caregiver sensitivity as a unidimensional measure (i.e., assessed caregiver sensitivity using a single scale), or a multidimensional measure (i.e., assessed caregiver sensitivity by combining multiple constructs).

### Maternal sensitivity and attachment: Previous reviews

Since Ainsworth and colleagues’ original study [10], several systematic reviews have been completed aiming to synthesize the literature examining the relationship between caregiver sensitivity and attachment [7, 21–25].

In the first synthesis of this body of literature, Goldsmith and Alansky [23] identified a small relationship between caregiver sensitivity and infant attachment. In contrast, a decade later, De Wolff and van IJzendoorn [7] updated this literature and identified a medium effect across studies examining maternal sensitivity and infant attachment. More recent reviews have replicated these findings, again reporting a medium effect for the relationship between sensitivity and infant attachment [21, 25]. Moderating effects have also been reported such that the strength of the relationship between maternal sensitivity and infant attachment was greater when infants were from middle class families compared to lower class families, or when infants were older at the time of the attachment assessment [7].

Syntheses have also been completed for the literature examining the relationship between caregiver sensitivity and attachment in children and adolescents [22, 24]. However, one of the studies [22] did not complete a meta-analytic synthesis and reviewed a combination of studies involving infant and child attachment. Whereas, the other study [24] completed a meta-analytic review of studies examining sensitivity and attachment from early childhood to adulthood, eliminating the preschool age. A gap in the literature exists in terms of the research specifically examining the relationship between caregiver sensitivity and preschool attachment. Furthermore, with approximately three decades of research since the inception of the Cassidy and Marvin [13] and Main and Cassidy [12] attachment coding systems, there is a wealth of literature to be synthesized in terms of the relationship between caregiver sensitivity and preschool attachment employing these systems. Additionally, given the parallels between Ainsworth et al.’s [10] original classification system and the preschool systems [12, 13], it will be important to meta-analytically investigate how the strength of the relationship between caregiver sensitivity and preschool attachment compares to past syntheses of caregiver sensitivity and infant attachment [7, 21, 23, 25]. Moreover, in order to maintain consistency and comparability to the previous related meta-analytic reviews noted above, the present review also implemented several relevant moderator variables (e.g., normative vs. clinical/risk populations, child age, child gender, socioeconomic status) to determine how these factors may impact the strength of the relationship between caregiver sensitivity and preschool attachment.

### The current study

The overarching aim of the present study was to synthesize and meta-analyze the literature examining the concurrent and longitudinal relationship between caregiver sensitivity and preschool attachment measured using the Cassidy and Marvin [13] and Main and Cassidy [12] coding systems. Given the heterogeneity in measurement of caregiver sensitivity, the literature was subdivided by the operationalization of caregiver sensitivity. Specifically, studies were either identified as employing a unidimensional measure of caregiver sensitivity (e.g., examining one aspect of caregiver sensitivity using a single rating of caregiver sensitivity), or a multidimensional measure of caregiver sensitivity (e.g., examining several aspects of the sensitivity of a caregiver by combining multiple ratings such as sensitivity, intrusiveness, warmth, etc.). Additionally, the effect of moderator variables on the longitudinal and concurrent relationship between caregiver sensitivity and preschool attachment was examined through meta-regression analyses. Consistent with previous related meta-analyses of caregiver sensitivity and child attachment [7, 21, 24, 25], moderator variables included sample demographics (e.g., normative vs. clinical/risk populations, child age, child gender, socioeconomic status) and study quality.

In accordance with the past literature, we predicted to identify a medium effect between caregiver sensitivity and preschool attachment. Additionally, we predicted that the effect sizes would be relatively larger when caregiver sensitivity was measured proximally closer to the measurement of preschool attachment (e.g., concurrent associations) compared to when caregiver sensitivity was measured at an earlier developmental period in relation to preschool attachment (e.g., longitudinal associations). Furthermore, owing to the fact that a multidimensional measure of caregiver sensitivity would encompass a greater number of aspects of caregiver sensitivity (e.g., nonintrusiveness, warmth, etc.), we predicted that the association between caregiver sensitivity and preschool attachment would have a relatively smaller effect size for the literature employing a unidimensional measure of caregiver sensitivity versus a multidimensional measure of caregiver sensitivity. In terms of the implemented moderator variables, we predicted that studies with greater age at assessment of attachment, middle/high socioeconomic status, normative samples, and a higher quality would be associated with a stronger relationship between caregiver sensitivity and preschool attachment. We did not have specific predictions for the moderating effect of gender given the lack of evidence for the moderating effect of this variable in previous related meta-analyses [7, 24]. However, we chose to include child gender in the moderator analyses due to past associations that have been identified between child gender with both maternal sensitivity and preschool attachment [26].

## Methods

### Search strategy

A systematic electronic literature search was completed with the assistance of an academic librarian from the Hospital for Sick Children, Toronto, Ontario, Canada. The search was conducted using four different electronic search engines (Medline, Embase, PsycINFO, and CINAHL), and was last updated on April 20, 2020. To facilitate a broad search from inception, there were no initial limitations on language or publication date. Search terms were identified through key terms related to the Preschool Attachment Classification System (PACS; [13]) and key terms within the title and abstracts of relevant articles employing the classification systems for coding preschool attachment [12, 13]. Search terms were systematically paired that were related to the construct of *attachment*, the classification systems for coding preschool attachment [12, 13], and children between 2–7 years of age. Search terms and pairings for the PsycINFO electronic search engine are provided in S1 Appendix.

The present review followed an a priori protocol using the Preferred Reporting Items for Systematic Reviews and Meta-Analyses (PRISMA guidelines; [27]). Review protocol was registered before data extraction on the PROSPERO Website (Registration Number CRD42017073417; [28]). The PRISMA checklist is provided in S2 Appendix.

### Inclusion/Exclusion criteria

Studies were included if they a) measured caregiver sensitivity, b) measured preschool attachment through coding attachment using the specified Main and Cassidy [12] and Cassidy and Marvin [13] preschool attachment classification systems among children who were over 2 years and up to 7 years of age, and c) examined the concurrent or longitudinal relationship between caregiver sensitivity and preschool attachment. Abstracts that did not clearly identify either the age at which attachment was measured or the type of measurement used to examine attachment were set aside for full-text review if: 1) they were authored by individuals identified to contribute to the development of the PACS manual [13]; 2) key authors in the field of child attachment; and 3) studies completed using National Institute of Child Health and Development (NICHD) data (see S3 Appendix for protocol for ambiguous abstracts).

Studies were excluded if they were not in English or French. Studies were also excluded if they were published pre-1985 because 1985 was the earliest documented reference to the Main and Cassidy preschool coding system [29]. Moreover, studies were excluded if they examined nonhuman attachment, did not examine attachment, examined attachment with children outside of the required age range (i.e., less than or equal to 2 years of age or older than 7 years of age). We excluded studies with children age 2 or below as this age falls outside the specified age range for coding attachment using the Cassidy and Marvin [13] system, and older than 7 as this age falls outside the specified age range for coding attachment using the Main and Cassidy [12] system. Moreover, studies were excluded if they were review articles, commentaries, abstracts, case studies, or dissertations. Articles examining attachment with children in the specified age range were excluded if they measured preschool attachment using a different procedure (e.g., Attachment Story Completion Task; [30] or employed a different coding system (e.g., Preschool Assessment of Attachment [PAA]; [31]). The decision to exclude the PAA coding system was based on the low correlation identified between Cassidy and Marvin’s [13] and Crittenden’s [31] preschool coding systems [32].

### Study selection

**Fig 1:**
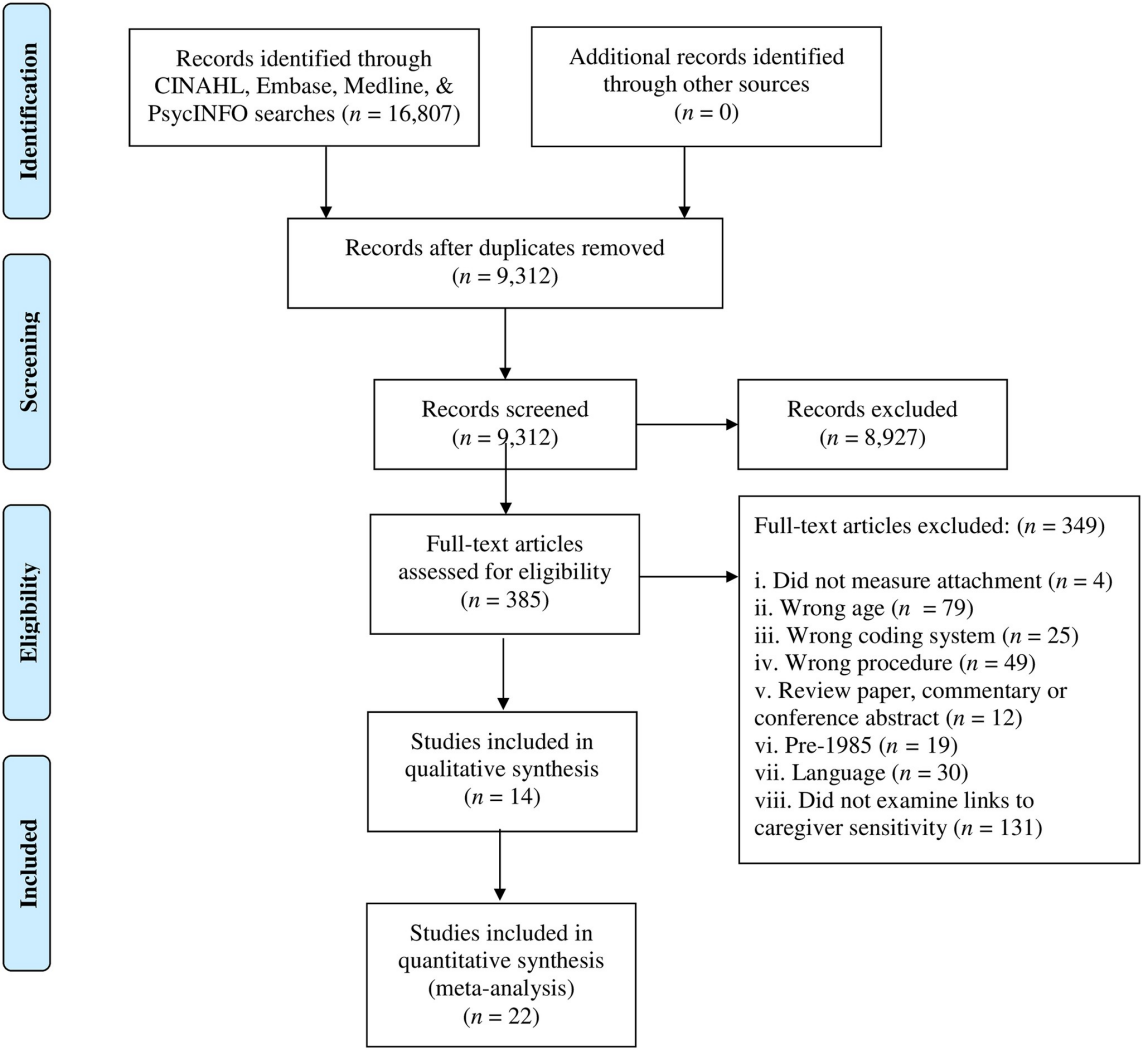
PRISMA flow diagram.

The systematic electronic literature search yielded a total of 16, 807 abstracts. The lead author and senior author designed the abstract selection criteria. After removing duplicates, the electronic search identified 9, 312 articles. Four independent reviewers screened the titles and abstracts that were included or excluded in accordance with the a priori selection criteria. Thirty-two percent of the abstracts were double-screened, with 88% to 98% of agreement between pairs of reviewers. Any discrepancy in inclusion/exclusion decisions was resolved through consensus. The full-text review yielded a total of 36 articles made up of 21 samples (*N* = 3, 847) that examined the concurrent and/or longitudinal relationship between caregiver sensitivity and preschool attachment measured using the pre-specified coding systems [12, 13]. The PRISMA Flow Diagram (Fig 1) presents the process of inclusion and exclusion of abstracts from the inception of the search to the final texts examined in the present study.

### Data extraction

Four reviewers independently completed the data extraction using a standardized extraction form and corresponding manual developed by the lead authors of this publication. One-hundred percent of the articles were double extracted by the lead author, and any discrepancies were resolved through weekly consensus meetings. The data extraction included publication year, demographic information (i.e., country, ethnicity, sample size, percentage of male children, mean years of age that preschool attachment was assessed, socioeconomic status, and clinical/risk vs. normative sample), methodology (i.e., type of caregiver sensitivity assessed [unidimensional, multidimensional, or both]), and when preschool attachment was analyzed in relation to caregiver sensitivity (i.e., concurrent, longitudinal, or both). Reliability statistics for the measurement of each of the relevant variables (i.e., caregiver sensitivity, preschoolers’ attachment) were also extracted in order to obtain the necessary data to calculate the attenuated effect sizes that account for variability in reliability coding across studies [33]. Statistical results were extracted from each study in order to calculate the effect size for differences in caregiver sensitivity as a function of secure-insecure or organized-disorganized preschool attachment. Authors were contacted if an article did not provide enough statistical information to be included in the meta-analyses. In instances where authors did not respond, or were not able to provide the requested statistical information, the article was synthesized qualitatively so as to not completely lose the information provided in that article.

### Quality assessment

There is currently no gold-standard measure available for examining the quality of observational studies [34]. Accordingly, the methodological quality of each study included in the present systematic literature review was assessed using a checklist adapted from the National Heart, Lung, and Blood Institute’s Quality Assessment Tool for Observational Cohort and Cross-Sectional Studies [35], Downs and Black [36], and Crombie [37]. See S4 Appendix for the checklist employed in the present review. The implementation of multiple tools for the quality assessment facilitated a hybrid approach to examining both a summary judgment checklist resulting in a total quantitative quality score, as well as the preferred method of a smaller checklist that focuses on the few main “potential sources of bias” [34] resulting in a more nuanced qualitative quality judgment. Two independent reviewers completed the quality assessment using the 16-item checklist and the overall quality judgment of all of the articles. There were few discrepancies between coders (Percentage agreement = 86%), which were resolved through consensus.

The modified checklist consisted of 16 items which were recorded as “yes” if the article fulfilled the requirement of the item, “no” if the article did not fulfill the requirement of the item, or “not applicable” in the rare case that the item did not correspond with a given article. A total quality score was calculated by determining the percentage of items that the study fulfilled out of the total applicable 16 items. A higher score was indicative of higher quality in a particular study.

In accordance with the NIH recommendations [35], six of the 16 items were identified as essential to determining overall quality judgment of the article (High vs. Low Quality). The items examined to determine overall quality pertained to: sample size and power; clearly defined, valid and reliable implementation of the predictor and outcome variables; coders of preschool attachment were blind to other study variables; > or equal to 80% retention in longitudinal studies; and accounting/controlling for potential confounding variables. Based on the aforementioned factors, each study was assigned an overall “Higher” or “Lower” quality judgment.

### Calculation of effect sizes and data analysis

Results were synthesized by first categorizing studies according to whether they examined the longitudinal or concurrent relationship between caregiver sensitivity and preschool attachment. Results were then subcategorized according to the operationalization of the caregiver sensitivity variable (i.e., unidimensional and/or multidimensional) and then again subdivided according to the preschool attachment outcome variable (e.g., secure vs. insecure and/or organized vs. disorganized). Study articles which reported sufficient statistical information to meta-analyze were included in the quantitative synthesis of the present review. This resulted in eight primary meta-analyses. Studies that did not provide sufficient statistical information to be included in the meta-analysis were synthesized qualitatively.

In instances where multiple studies reported on the same sample, the study that was most comparable to the other studies (e.g., similar operationalization of caregiver sensitivity, completing the analysis with a secure-insecure/organized-disorganized dichotomy rather than using a rating scale, mean years of age that preschool attachment was assessed) was prioritized for quantitative synthesis. If studies were drawn from the same sample and were equal in all aspects, the study with the full (larger) sample size was included. A similar approach was taken for studies that reported multiple statistical tests with the same variables, such that efforts were made to use the full sample and select the test that utilized variables *most* similar to the other studies in the given quantitative synthesis. Of note, some longitudinal studies consisted of multiple time points in which caregiver sensitivity was assessed in relation to subsequent measures of preschool attachment. In these instances, the child age at assessment of caregiver sensitivity that was most consistent with other studies contained within a given meta-analysis were selected, while also considering the above noted factors (i.e., similar operationalizations of caregiver sensitivity).

#### Quantitative synthesis

The standardized mean difference effect sizes were calculated for studies that provided sufficient data to be included in the relevant quantitative synthesis. First Cohen’s *d* effect size was calculated and then it was converted to Hedges’ *g*, because Hedges’ *g* corrects for a slight small sample bias that has been shown in Cohen’s *d* [38]. After Hedges’ *g* calculations were completed, eight separate meta-analyses were run through random-effects models using the metaphor R package [39] in RStudio (Version 3.6.0). The meta-analysis dataset is provided in S5 Appendix. The completed analyses were examined for the overall effect size (Hedges’ *g*), significance level, and corresponding 95% confidence intervals. Based on the assertion that Cohen’s [40, 41] traditional categorizations for effect sizes are too stringent, new guidelines for interpretation of effect sizes [42] were implemented for interpreting effect sizes in the present review. New recommendations for interpreting effect sizes were previously presented using Pearson’s *r* [42], and were therefore converted to Cohen’s *d* for adequate interpretation of the present meta-analyses. Recommended categorizations were: very small effect (*r* = .05 or *d* = .10), small effect (*r* = .10 or *d* = .20), medium effect (*r* = .20 or *d* = .40), large effect (*r* = .30 or *d* = .62), and very large effect (*r* = .40 or *d* = .87).

Heterogeneity among the studies was assessed using the *Q-*statistic which indicates if there is a statistically significant amount of heterogeneity between studies, and the *I*^*2*^-statistic indicates the size of heterogeneity (e.g., small [25%], medium [50%], large [75%]; [38]). An *I*^*2*^-statstic equal to 100% indicates that all of the variability is due to between study differences, whereas an *I*^*2*^-statstic of 0% indicates that all of the variability is due to sampling error [38]. Forest plots corresponding to each of the main meta-analyses were completed. Each forest plot illustrates the effect sizes and corresponding confidence intervals for each study included in a given meta-analysis. The center point visually depicts each study’s effect sizes (Hedges’ *g*) and confidence intervals. A square or bar crossing 0 is indicative of no difference in caregiver sensitivity among the attachment outcome (e.g., secure vs. insecure or organized vs. disorganized). Square points on the right side of 0 are indicative of higher caregiver sensitivity among caregivers who have secure versus insecure, or organized versus disorganized children.

Meta-regression analyses were also conducted in order to examine how potential moderators (e.g., quality score, child gender, child age, sample type [clinical vs. normative], socioeconomic status) moderate the longitudinal and/or concurrent relationship between caregiver sensitivity (i.e., either unidimensional or multidimensional) and preschool attachment (i.e., either secure vs. insecure or organized vs. disorganized).

#### Qualitative synthesis

Articles were qualitatively synthesized if insufficient statistical information was provided in the article to be included in the quantitative synthesis, or if the article was drawn from a sample that had already been entered into the relative quantitative synthesis. Qualitative articles were synthesized by reporting the magnitude (i.e., effect size) and direction of the study effects. Moderator variables examined in the quantitative synthesis were also considered in the qualitative synthesis, through consideration of the study characteristics and by examination of any covariates that were included in the analyses in the study articles.

## Results

### Studies included

The current review included 36 articles and 21 samples (*N* = 3, 847), with 22 of those articles being included in one of the eight primary meta-analyses. Studies included in the present review are marked with an asterisk within the references section and cited throughout the results section and relevant figures and tables. Of note, our initial abstract screening included articles written in French. However, during full-text review it was determined that all of the French articles were drawn from the same samples of English articles by the Moss research group that have been included in the present review. Therefore, all of the French articles were omitted for the present review.

### Study characteristics

An overview of the study characteristics is presented in Table 1.

**Table 1:** Study characteristics.

Research Group/Sample	Reference	Country	*N*^c^	Sample Type	Child Age at Caregiver Sensitivity Assessment for Studies with Longitudinal Analyses^d^	Child Age at Attachment Assessment	Attachment Categorizations	Caregiver Sensitivity Measure(s)	Caregiver Sensitivity Composition	Quality Score (Quality Judgment)^f^
Bureau	[16]	Canada	107	Normative		3.89 years	SI	Modified Parent-Child Interaction Scale	Unidimensional	80.00 (Higher)
Bureau	[15]	Canada	107	Normative		3.89 years	SI	Modified Parent-Child Interaction Scale	Multidimensional	66.67 (Higher)
							OD			
Bureau	[17]	Canada	144	Normative		3.91 years	SI	Modified Parent-Child Interaction Scale	Unidimensional	86.67 (Higher)
							OD			
MAVAN	[56]	Canada	159	Clinical/Risk	6 months	3 years	SI	Ainsworth Scales	Multidimensional	50.00 (Lower)
							OD			
MAVAN	[58]	Canada	301	Clinical/Risk	6 months	3 years	OD	Ainsworth Scales	Multidimensional	75.00 (Higher)
McElwain	[71]	USA	120	Normative		2.73 years	SI	Author Developed	Multidimensional	71.43 (Higher)
McElwain	[72]	USA	127	Normative		2.73 years	SI	Author Developed	Multidimensional	85.71 (Higher)
McElwain	[74]	USA	127	Normative		2.73 years	SI	Author Developed	Multidimensional	57.14 (Lower)
Moss^a^	[63]	Canada	121	Normative		6.25 years	SI	Parent-Child Interaction Scale	Unidimensional	56.25 (Lower)
							OD			
Moss^a^	[64]	Canada	111	Normative		6.3 years	SI	Parent-Child Interaction Scale	Unidimensional	56.25 (Lower)
							OD			
Moss^b^	[61]	Canada	83	Normative		5.56 years	SI	Author Developed	Unidimensional	62.50 (Higher)
							OD			
Moss^b^	[62]	Canada	151	Normative		3.67 years	SI	Parent-Child Interaction Scale	Unidimensional	66.67 (Higher)
							OD			
Moss^b^	[47]	Canada	120	Normative	3.67 years	3.67 years and 5.58 years	SI	Parent-Child Interaction Scale	Unidimensional	75.00 (Higher)
							OD			
Moss^a,b^	[48]	Canada	242	Normative	4 years	6 years	SI	Parent-Child Interaction Scale	Unidimensional	81.25 (Higher)
							OD			
NICHD SECCYD	[43]	USA	1077	Normative	2 years	3 years	SI	NICHD SECCYD	Multidimensional	87.50 (Higher)
NICHD SECCYD	[54]	USA	1060	Normative	1.69 years^g^	3 years	SI	NICHD SECCYD	Multidimensional	75.00 (Lower)
NICHD SECCYD	[55]	USA	1140	Normative	2 years	3 years	SI	NICHD SECCYD	Multidimensional	75.00 (Lower)
							OD			
NICHD SECCYD	[26]	USA	1140	Normative	1.69 years^g^	3 years	SI	NICHD SECCYD	Multidimensional	87.50 (Higher)
							OD			
NICHD SECCYD	[65]	USA	1140	Normative		3 years	SI	NICHD SECCYD	Unidimensional	75.00 (Higher)
							OD			
NICHD SECCYD	[57]	USA	303	Normative	1.69 years^g^	3 years	SI ^e^	NICHD SECCYD	Multidimensional	56.25 (Lower)
							OD ^e^			
NICHD SECCYD	[45]	USA	1016	Normative		3 years	SI	NICHD SECCYD	Multidimensional	87.50 (Lower)
Unique	[59]	USA	69	Clinical/Risk		4.6 years	SI	Author Developed	Unidimensional	62.50 (Higher)
							OD			
Unique	[52]	USA	69	Clinical/Risk	1.88 years^h^	2.50 years	SI	ORCE	Multidimensional	81.25 (Higher)
Unique	[32]	USA	51	Clinical/Risk	9 months	3.25 years	SI	CARE-Index	Unidimensional	37.50 (Lower)
							OD			
Unique	[69]	Netherlands	59	Clinical/Risk		4.75 years	SI	NICHD SECCYD	Multidimensional	53.33 (Lower)
							OD			
Unique	[60]	USA	74	Clinical/Risk		4.40 years	SI	Author Developed	Unidimensional	62.50 (Lower)
Unique	[70]	Ukraine	64	Clinical/Risk		4.24 years	SI	EAS	Multidimensional	66.67 (Higher)
							OD			
Unique	[44]	Israel	40	Clinical/Risk		3.94 years	SI	EAS	Unidimensional	87.50 (Higher)
							OD			
Unique	[53]	USA	82	Clinical/Risk	9 months	4 years	SI	Author Developed	Multidimensional	68.75 (Lower)
Unique	[46]	England	128	Normative	8 months	4.29 years	SI	Ainsworth Scales	Unidimensional	75.00 (Higher)
Unique	[73]	United Kingdom	129	Clinical/Risk		4 years	SI	Author Developed	Multidimensional	43.75 (Lower)
							OD^e^			
Unique	[66]	USA	29	Clinical/Risk		3.92 years	SI	Ainsworth Scales	Unidimensional	56.25 (Lower)
Unique	[49]	USA	732	Clinical/Risk		3 years	SI	Author Developed	Unidimensional	56.25 (Lower)
							OD			
Unique	[67]	United Kingdom	98	Normative		4.5 years	SI	Author Developed	Both	75.00 (Lower)
							OD			
Unique	[50]	England	78	Normative	3.5 years	4.5 years	SI	Author Developed	Both	60.00 (Lower)
							OD			
Unique	[68]	Canada	161	Clinical/Risk		2.88 years	OD	EAS	Unidimensional	68.75 (Higher)

NICHD SECCYD = National Institute for Child Development: Study of Early Child Care and Youth Development; MAVAN = Maternal Adversity, Vulnerability and Neurodevelopment; EAS = Emotional Availability Scales; ORCE = Observational Record of the Caregiving Environment; SI = Secure-Insecure; OD = Organized-Disorganized.

^a^ Moss Research Group First Cohort.

^b^ Moss Research Group Second Cohort.

^a,b^ Moss Research Group First and Second Cohort Combined.

^c^ Study sample size reflective of that used in the present quantitative and qualitative analysis.

^d^ Child age at assessment of caregiver sensitivity is provided for only the longitudinal analyses within the current review. For studies within the current review to be considered concurrent it was required that caregiver sensitivity and preschool attachment were assessed within 1 month or less than 1 month of one another and therefore the child age at assessment of caregiver sensitivity for concurrent study analyses are available in the subsequent column denoting child age at assessment of attachment.

^e^ Interpretation of dichotomy based on non-significant findings for caregiver sensitivity as a function of the four categories of attachment, as insufficient information was available to interpret the two-way dichotomy.

^f^The overall quality judgment (higher vs. lower) is determined based on meeting six key criteria (sample size and power; clearly defined, valid and reliable implementation of the predictor and outcome variables; coders of preschool attachment blind to other study variables; > or equal to 80% retention in longitudinal studies, accounting/controlling for potential confounding variables) from the 16 criteria used to determine the quality score.

^g^ Sensitivity averaged over 6, 15, 24, and 36 month time points.

^h^ Sensitivity averaged over 15 and 30 month time points.

#### Demographics

The majority of the study articles were conducted in the United States (*k* = 17) and Canada (*k* = 12), with the remaining studies occurring in Europe (*k* = 6) and Israel (*k* = 1). Several of the studies were drawn from the same samples, owing to multiple publications by the same research group: Bureau research group (*k* = 3), McElwain research group (*k* = 3), the Maternal Adversity, Vulnerability and Neurodevelopment research group (*k* = 2), and the National Institute for Child Development: Study of Early Child Care and Youth Development (NICHD SECCYD; *k* = 7). Several studies were also completed by the Moss research group, which consisted of studies from an earlier cohort (*k* = 2) and a later cohort (*k* = 3), and a study which collapsed the two cohorts (*k* = 1). Overall, there were a total of 21 samples among all of the studies. Almost half of the studies (*k* = 15) were drawn from a unique sample in the present review. The majority of the studies were identified as coming from a normative sample (*k* = 22) and Middle/High socioeconomic status (*k* = 29). Most of the children were between 2- to 5-years-old when they participated in the modified separation-reunion procedure.

#### Caregiver sensitivity

Approximately half of the studies (*k* = 17) were identified as operationalizing caregiver sensitivity as a unidimensional measure and half were identified as operationalizing caregiver sensitivity as a multidimensional measure (*k* = 19). Two of these studies examined the relationship between caregiver sensitivity and preschool attachment through employing both a unidimensional and multidimensional measure of caregiver sensitivity.

#### Attachment categorizations

Given a priori knowledge that studies varied in their categorizations of preschool attachment outcomes [14], study results were extracted for secure-insecure or organized-disorganized preschool attachments outcomes, or the necessary statistics were extracted to calculate outcomes in terms of these dichotomies (i.e., collapsing means and standard deviations of caregiver sensitivity for A/C/D vs. B, converting the correlation between caregiver sensitivity and a security scale to a mean difference effect size). Of note, studies identified as examining the relationship between caregiver sensitivity and the controlling attachment categories (i.e., controlling-caregiving, controlling-punitive) were included within the organized-disorganized quantitative and qualitative syntheses throughout the current review and are referred to as the organized-disorganized dichotomy outcomes herein. Overall, almost all of the studies were interpretable in terms of a secure-insecure dichotomy (*k* = 33), and most of the studies were interpretable in terms of an organized-disorganized dichotomy (*k* = 24).

#### Quality

**Fig 2:**
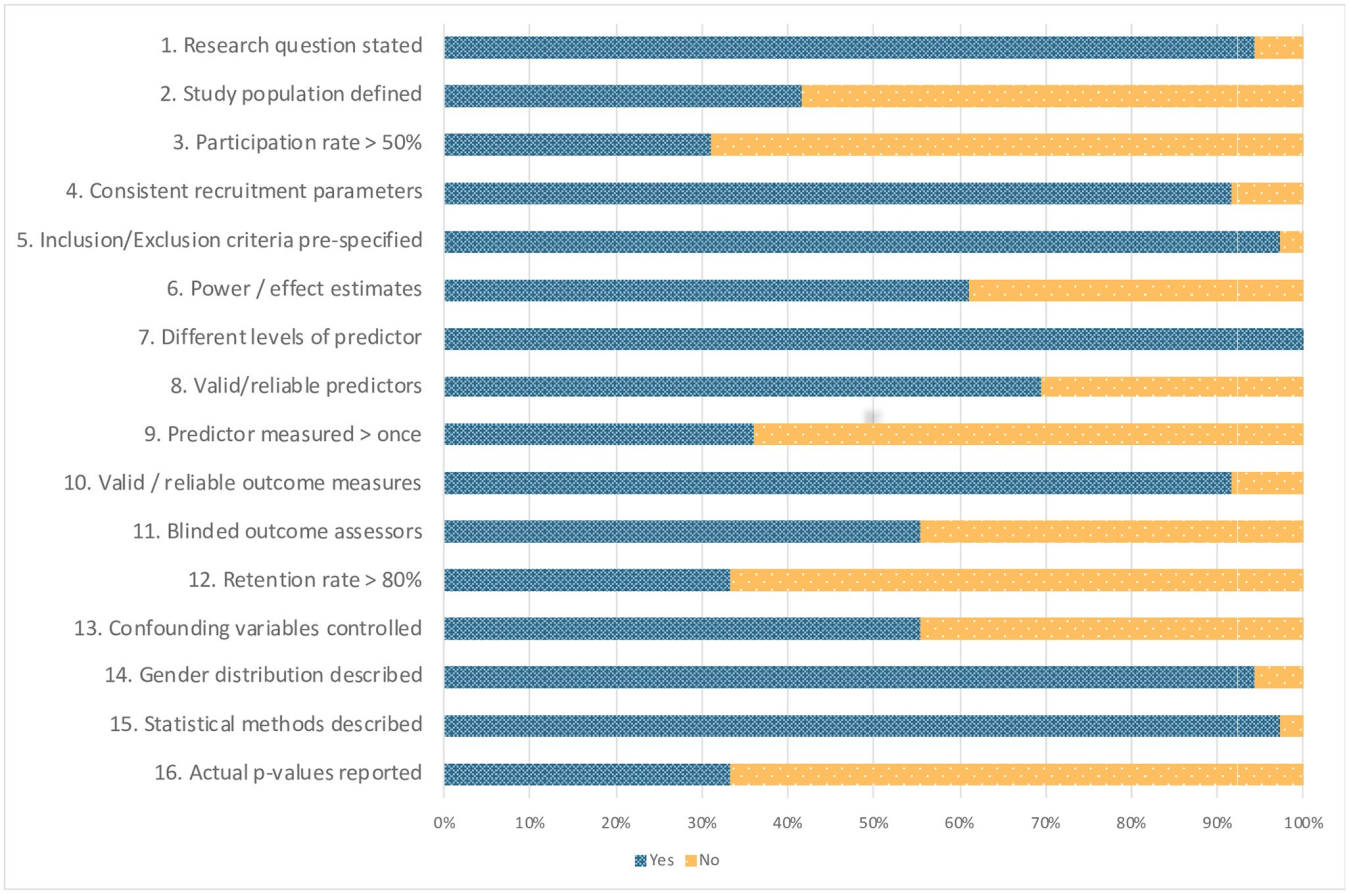
Quality assessment scores. Percentage of studies that fulfilled the criteria for each item on the quality assessment checklist.

The mean quality score for the 36 studies was 71.90%. The lowest quality score was 37.50% [32] and the highest quality score was 87.50% [26, 43–45]. Fig 2 provides a visual depiction of the percentage of studies that fulfilled each of the 16 criteria in the quality assessment that made up the total score. In terms of the six criteria that contributed to the overall quality judgment, 61.1% of the studies provided a power analysis or effect size estimates, 55.6% reported that potential confounding variables were assessed and adjusted for, and 69.4% and 91.7% provided clear, valid, and reliable information about the predictor (caregiver sensitivity) and outcome variables (preschool attachment), respectively. Approximately half (55.6%) of the studies reported that attachment coders were blind to the other study variables. In contrast, few studies (33.3%), reported that retention rate of participants in longitudinal studies was 80% or greater. Approximately half of the studies (*k* = 17) were given a higher quality judgment, and the remaining (*k* = 19) were given a lower quality judgement.

### Quantitative and qualitative syntheses

The following sections present the quantitative and qualitative syntheses for the current review. See Table 2 for a summary of the quantitative and qualitative syntheses.

**Table 2:** Quantitative and qualitative summaries.

	Articles analyzed	Synthesis technique	Effect size	Summary of results
1. Longitudinal relationship between caregiver sensitivity and preschool attachment				
1.1. Unidimensional caregiver sensitivity and preschool attachment				
1.1.1. *Secure vs*. *Insecure*	[46,48,50]	Quantitative	Medium effect (*g* = 0.46)	Medium effect indicating higher levels of unidimensional caregiver sensitivity among caregivers of secure relative to insecure children (*g* = 0.46, *p* = .002, 95% CI [0.17, 0.75]). Higher effects among samples where children were older when preschool attachment was assessed.
1.1.2. *Secure vs*. *Insecure*	[47,49]	Qualitative	Mixed effects	One study reported a large overall effect (*g* = .0.84) suggesting that caregiver sensitivity was higher for caregivers of children who were secure versus insecure. The other study reported that caregiver sensitivity did not significantly differ as a function of child secure and insecure attachment. Of note, the study reporting a non-significant finding controlled for both child birthweight and socioeconomic status.
1.1.3. *Organized vs*. *Disorganized/Controlling*	[48,50]	Quantitative	Medium effect (*g* = 0.51)	Medium effect indicating higher levels of unidimensional caregiver sensitivity among caregivers of organized relative to disorganized children (*g* = 0.51, *p* = .08, 95% CI [-0.06, 1.09]).
1.1.4. *Organized vs*. *Disorganized/Controlling*	[47,49]	Qualitative	Mixed effects	One study revealed a medium effect (*g* = .42) suggesting that unidimensional caregiver sensitivity was higher for caregivers of children who were organized versus disorganized. The other study reported that caregiver sensitivity did not significantly differ as a function of child organization versus disorganization. Of note, the study reporting a non-significant finding controlled for both child birthweight and socioeconomic status.
1.2. Multidimensional caregiver sensitivity and preschool attachment				
1.2.1. *Secure vs*. *Insecure*	[50,52,53,55,56]	Quantitative	Small effect (*g* = 0.34)	Small effect indicating higher multidimensional sensitivity levels of caregiver sensitivity among caregivers of secure relative to insecure children (*g* = 0.34, *p* = .004, 95% CI [0.11, 0.56]).
1.2.2. *Secure vs*. *Insecure*	[26,43,54,57]	Qualitative	Mixed effects	Four studies from the same sample (NICHD SECCYD) revealed a small (*g* = .32) to medium (*g* = .49) overall effect suggesting that multidimensional caregiver sensitivity was higher for caregivers of children who were secure versus insecure.
1.2.3. *Organized vs*. *Disorganized/Controlling*	[50,55,56]	Quantitative	Small effect (*g* = 0.39)	Small effect *g* = 0.39, *p* = .001, 95% CI [0.16, 0.62], indicating that higher multidimensional caregiver sensitivity among caregivers of organized children relative to disorganized children.
1.2.4. *Organized vs*. *Disorganized/Controlling*	[26,57,58]	Qualitative	Mixed effects	Two studies from the same sample revealed a small (*g* = .30) to medium (*g* = .47) effect, suggesting that multidimensional caregiver sensitivity is higher for caregivers of organized versus disorganized children. Another study reported a medium overall effect (*g* = .61), supporting the finding that multidimensional caregiver sensitivity is higher for caregivers of children who are organized relative to disorganized.
2. Concurrent relationship between caregiver sensitivity and preschool attachment				
2.1 Unidimensional caregiver sensitivity and preschool attachment				
2.1.1. *Secure vs*. *Insecure*	[16,44,50, 59,60,62,63,65,66,67]	Quantitative	Medium effect (*g* = 0.59)	Medium effect indicating higher unidimensional levels of caregiver sensitivity among caregivers of secure versus insecure children (*g* = 0.59, *p* < .0001, 95% CI [0.40, 0.79]).
2.1.2. *Secure vs*. *Insecure*	[17,32,47,48,49,61,64]	Qualitative	Mixed effects	In five studies, stemming from three different samples, there was a medium (*g* = .49) to very large (1.09) effect, suggesting that unidimensional caregiver sensitivity was higher for caregivers of children who were secure as opposed to insecure. Two studies reported a non-significant relationship between unidimensional caregiver sensitivity and preschool attachment, but these studies were noted to report on clinical samples of lower socioeconomic status and were judged to have lower quality.
2.1.3. *Organized vs*. *Disorganized/Controlling*	[17,44,50,59,62,63,65,67,68]	Quantitative	Medium effect (*g* = 0.50)	Medium effect indicating higher unidimensional levels of caregiver sensitivity for caregivers of organized children relative to disorganized children (*g* = 0.50, *p* < .0001, 95% CI [0.29, 0.72]).
2.1.4. *Organized vs*. *Disorganized/Controlling*	[32,47,48,49,61,64]	Qualitative	Mixed effects	In four studies comprised of two different samples, there was a medium (*g* = .42) to very large (*g* = 1.66) effect, suggesting that unidimensional caregiver sensitivity was higher for caregivers of children who were organized compared to disorganized. Two additional studies identified a non-significant relationship between unidimensional caregiver sensitivity and preschool attachment. The studies reporting non-significant findings were among clinical samples, of lower socioeconomic status, and were judged to have lower quality.
2.2 Multidimensional caregiver sensitivity and preschool attachment				
2.2.1. *Secure vs*. *Insecure*	[15,53,55,67,69,70,72]	Quantitative	Medium effect (*g* = 0.49)	Medium effect (*g* = 0.49, *p* < .0001, 95% CI [0.39, 0.59]) indicating higher levels of multidimensional caregiver sensitivity among caregivers with secure children relative to insecure children.
2.2.2. *Secure vs*. *Insecure*	[43,45,71,73,74]	Qualitative	Mixed effects	Four studies demonstrated small (*g* = .35), medium (*g* = .41 to .45), and large (*g* = .74) effects indicative that multidimensional caregiver sensitivity is higher among caregivers with secure children compared to insecure children. Another study, reported a non-significant relationship between multidimensional caregiver sensitivity and preschool attachment.
2.2.3 *Organized vs*. *Disorganized*	[15,55,67,69,70]	Quantitative	Small effect (*g* = 0.39)	Small effect indicating higher multidimensional levels of caregiver sensitivity among caregivers of organized children relative to disorganized children (*g* = 0.39, *p* < .0001, 95% CI [0.25, 0.53]).
2.2.4 *Organized vs*. *Disorganized*	[73]	Qualitative	Non-significant (no effect size reported)	One study reported a non-significant relationship between multidimensional caregiver sensitivity and preschool attachment.

### 1. Longitudinal relationship between caregiver sensitivity and preschool attachment

#### 1.1. Longitudinal associations between unidimensional caregiver sensitivity and preschool attachment

Five studies examined the longitudinal relationship between unidimensional caregiver sensitivity and preschool attachment [46–50].

**Fig 3:**
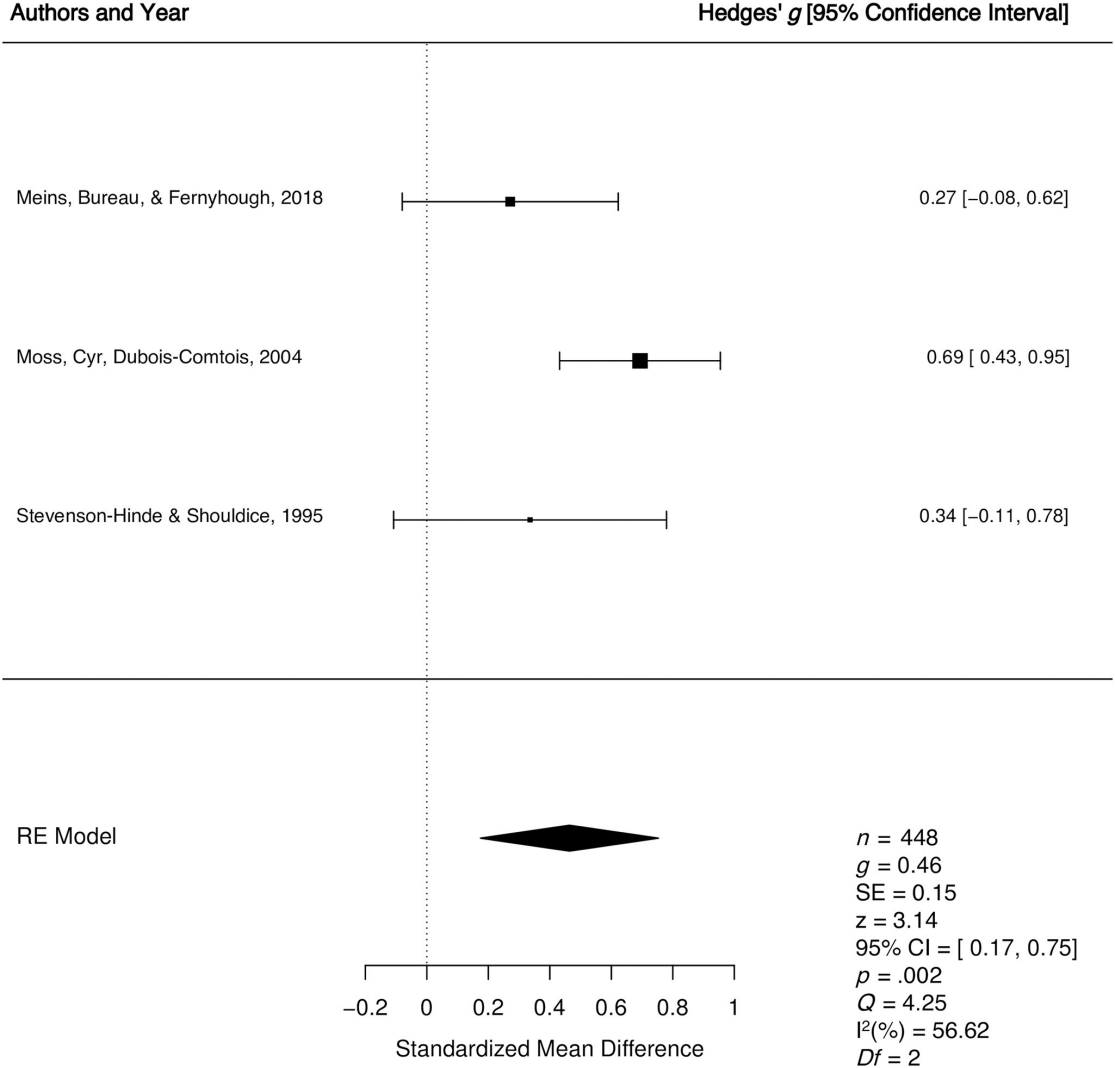
Forest plot for the longitudinal relationship between unidimensional caregiver sensitivity and secure versus insecure preschool attachment. RE = Random Effects Model; *g* = Hedges’ *g; Q* = Cochran’s heterogeneity statistic; I^2^ = percentage of variability across studies that is due to between-study heterogeneity.

*1*.*1*.*1*. *Secure vs*. *insecure*: *Quantitative synthesis*. Three studies were included in the quantitative synthesis examining differences in unidimensional caregiver sensitivity for secure versus insecure children. The weighted mean effect size of differences in unidimensional caregiver sensitivity for children who were secure versus insecure was calculated from a total sample of 448 child-caregiver dyads, that were all from normative samples. Two of the studies had been assigned a higher quality judgment [46, 48] and one of the studies had been assigned a lower quality judgment [50]. The meta-analysis revealed a medium effect *g* = 0.46, *p* = .002, 95% CI [0.17, 0.75], indicating higher levels of unidimensional caregiver sensitivity among secure versus insecure children (See Fig 3). There was a moderate degree of true between study heterogeneity (*Q* = 4.25, *p* = .12, *I*^2^ = 56.62%). The result of Egger’s regression test [51] for funnel plot asymmetry was non-significant (*p* = .13), suggesting no evidence of publication bias.

Three separate moderator analyses were conducted to determine if the longitudinal relationship between unidimensional sensitivity and secure versus insecure attachment varies as a function of key study variables. There was a significant effect of preschool attachment age (Q_b_ = 4.25, *p* = 0.04), indicating larger between-group differences for unidimensional caregiver sensitivity in samples where children were older (*g* = 0.24). The moderator analyses were non-significant for quality score (Q_b_ = 0.75, *p* = 0.38) and child gender (Q_b_ = .16, *p* = 0.68). Moderator analyses could not be conducted for sample type (clinical vs. normative) and socioeconomic status (low vs. middle/high) due to lack of variability in the studies (i.e., all samples were normative with a high/middle socioeconomic status).

*1*.*1*.*2*. *Secure vs*. *insecure*: *Qualitative synthesis*. Two studies were included in the qualitative synthesis examining differences in unidimensional caregiver sensitivity for secure versus insecure children [47, 49]. One of the studies [49] was not included in the quantitative synthesis due to reporting insufficient statistical information for the meta-analysis. The other study [47] was drawn from the same sample as a study [48] that was prioritized for quantitative synthesis.

For the present qualitative synthesis, one study [49] was from a clinical sample and assigned a lower quality judgement, and the other study was from a normative sample [47] and assigned a higher quality judgment. One study [47] examined the longitudinal relationship between caregiver sensitivity and secure versus insecure preschool attachment. Means, standard deviation and sample sizes were pooled to combine secure groups and insecure groups, and the between-group effect size was calculated in order to assess the direction and magnitude of the differences. There was a large overall effect (*g* = 0.84) suggesting that caregiver sensitivity was higher for caregivers of children who were secure compared to insecure. In the study using a clinical sample [49], the longitudinal relationship between caregiver sensitivity and preschool attachment was non-significant, such that caregiver sensitivity did not differ among caregivers of children who were secure versus insecure. It is important to note that, unlike the studies included in the quantitative synthesis, this study controlled for both the child’s birthweight and the socioeconomic status of the family.

**Fig 4:**
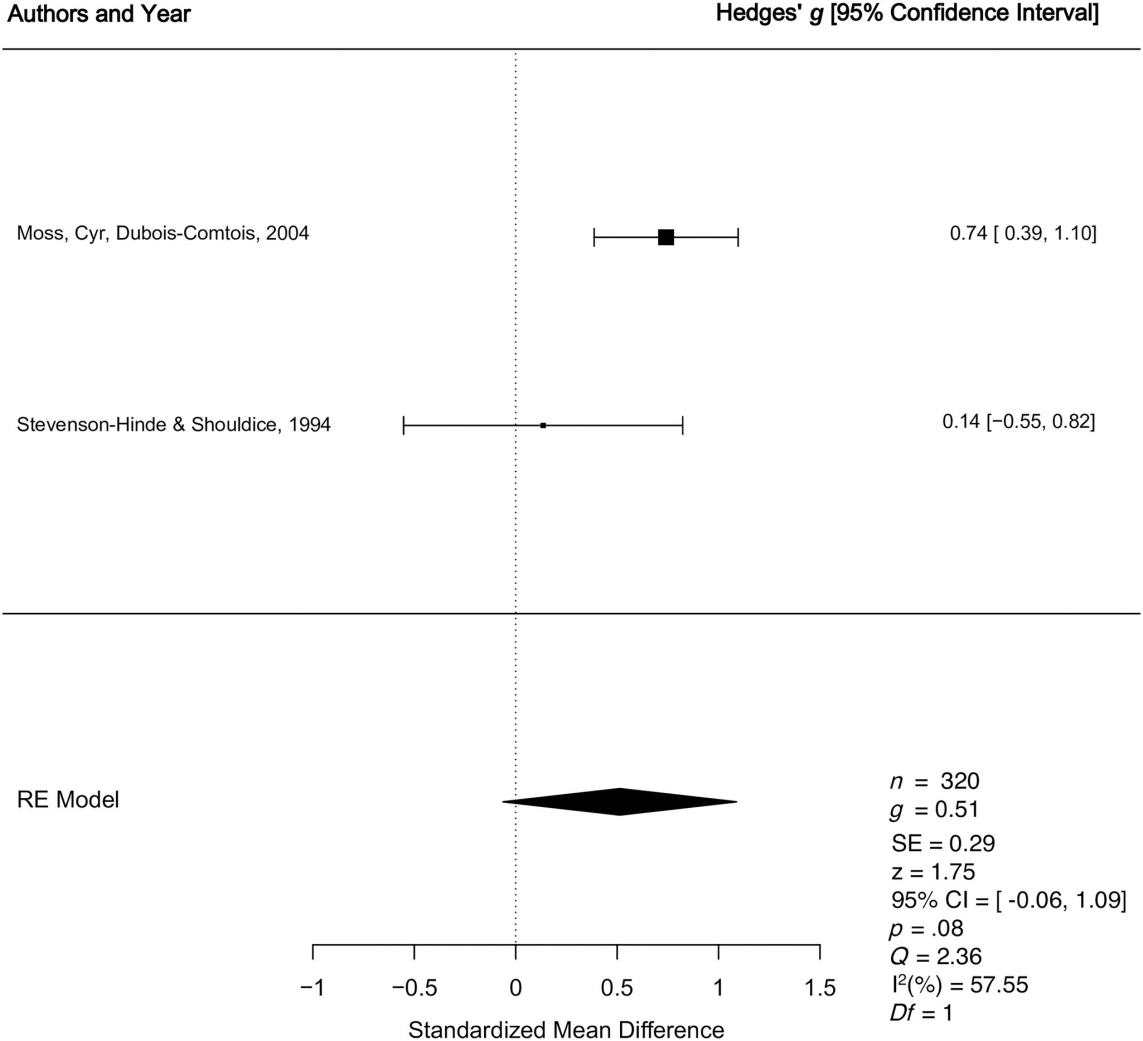
Forest plot for the longitudinal relationship between unidimensional caregiver sensitivity and organized versus disorganized preschool attachment. RE = Random Effects Model; *g* = Hedges’ *g; Q* = Cochran’s heterogeneity statistic; I^2^ = percentage of variability across studies that is due to between-study heterogeneity.

*1*.*1*.*3*. *Organized vs*. *disorganized*: *Quantitative synthesis*. Two studies were included in the quantitative synthesis examining differences in unidimensional caregiver sensitivity for organized versus disorganized children. The weighted mean effect size of differences in unidimensional caregiver sensitivity for children who were organized versus disorganized was calculated from a total sample of 320 child-caregiver dyads drawn from normative samples. One study [48] was assigned a higher quality judgment and one study [50] was assigned a lower quality judgment. The meta-analysis revealed a medium effect *g* = 0.51, *p* = .08, 95% CI [-0.06, 1.09], indicating higher levels of unidimensional caregiver sensitivity among caregivers of organized versus disorganized children (See Fig 4). There was a moderate degree of true between study heterogeneity (*Q* = 2.36, *p* = .12, *I*^2^ = 57.55%). Due to only having two studies included in the meta-analysis, it was not possible to complete Egger’s regression test [51] for funnel plot asymmetry. Additionally, because there were only two studies included in the meta-analysis, it was not possible to conduct moderator analyses to determine if the relationship between unidimensional sensitivity and organized versus disorganized attachment varied as a function of key study variables (e.g., quality score, child gender, sample type [clinical vs. normative], socioeconomic status, or age at preschool attachment).

*1*.*1*.*4*. *Organized vs*. *disorganized*: *Qualitative synthesis*. Two studies were included in the qualitative synthesis examining differences in unidimensional caregiver sensitivity for organized versus disorganized children [47, 49]. One of the studies [49] was not included in the quantitative synthesis due to reporting insufficient statistical information for the meta-analysis. The other study [47] was drawn from the same sample as a study [48] that was prioritized for quantitative synthesis.

For the present qualitative synthesis, one study [49] was from a clinical sample and assigned a lower quality judgement, and the other study was from a normative sample [47] and assigned a higher quality judgment. One study [47] examined the longitudinal relationship between caregiver sensitivity and organized versus disorganized preschool attachment. Means, standard deviations and sample sizes were pooled to combine organized groups in order to compare the organized group with the disorganized group by calculating the between-group effect size to assess the direction and magnitude of the differences. There was a medium overall effect (*g* = 0.42) suggesting that caregiver sensitivity was higher for caregivers of children who were organized compared to disorganized. In the study using a clinical sample [49], the longitudinal relationship between caregiver sensitivity and preschool attachment was non-significant, such that caregiver sensitivity did not differ among caregivers of children who were organized versus disorganized. It is important to note that, unlike the studies included in the quantitative synthesis, this study controlled for both the child’s birthweight and the socioeconomic status of the family.

#### 1.2. Longitudinal associations between multidimensional caregiver sensitivity and preschool attachment

Ten studies examined the longitudinal relationship between multidimensional caregiver sensitivity and preschool attachment [26, 43, 50, 52–58].

**Fig 5:**
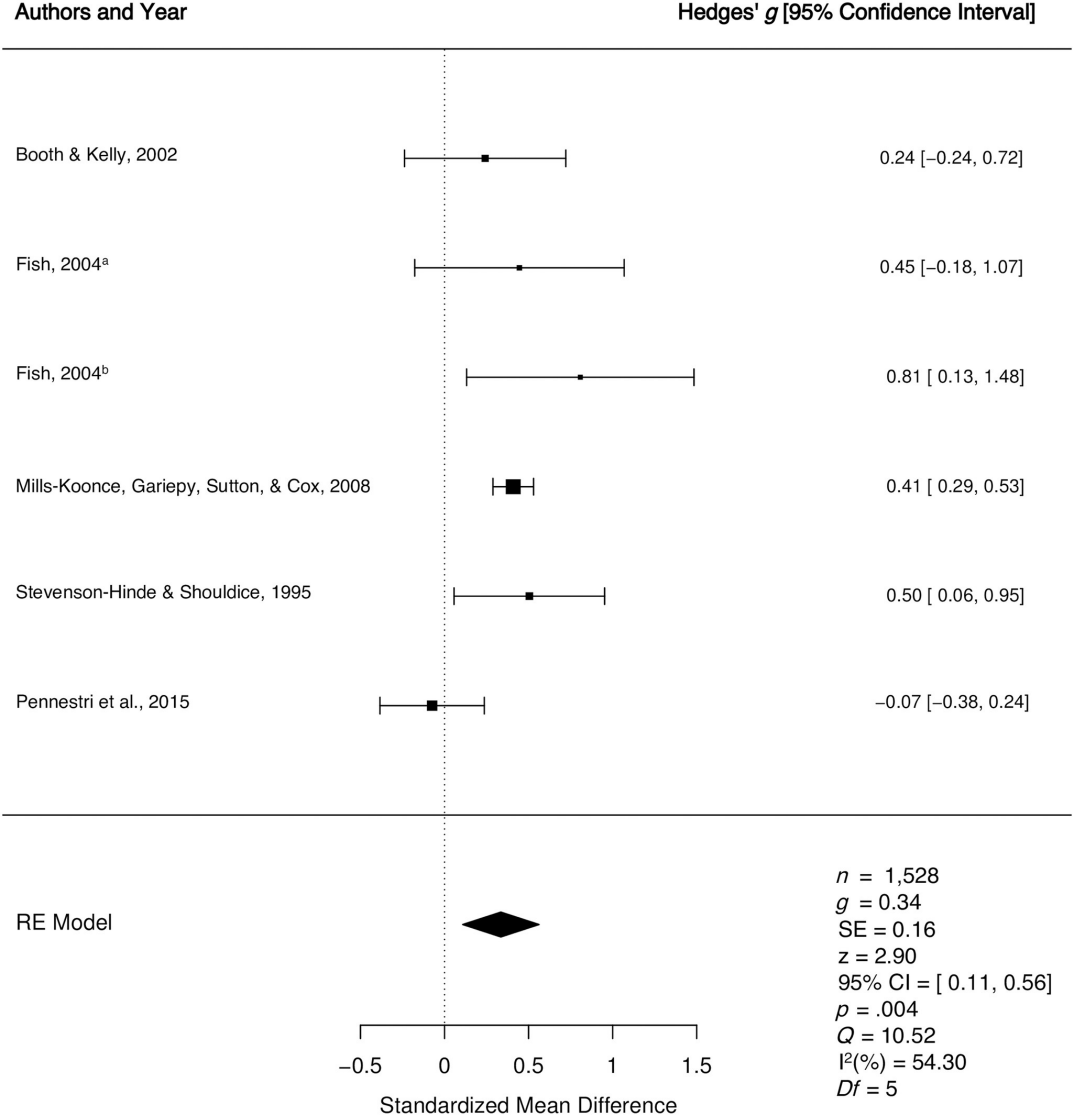
Forest plot for the longitudinal relationship between multidimensional caregiver sensitivity and secure versus insecure preschool attachment. RE = Random Effects Model; *g* = Hedges’ *g; Q* = Cochran’s heterogeneity statistic; I^2^ = percentage of variability across studies that is due to between-study heterogeneity.

*1*.*2*.*1*. *Secure vs*. *insecure*: *Quantitative synthesis*. Five studies were included in the quantitative synthesis examining differences in multidimensional caregiver sensitivity for secure versus insecure children. It is important to note that among the five studies, one study was treated as two separate studies and entered twice [53], because separate analyses were run for children with a secure and insecure infant history and the necessary statistical information to combine these effects to enter it as one study was not available. The weighted mean effect size of differences in multidimensional caregiver sensitivity for children who were secure versus insecure was calculated from a total of 1, 528 child-caregiver dyads, that consisted of three clinical samples [52, 53, 56] and two [50, 55] normative samples. One study [52] had been assigned a higher quality judgment and four studies [50, 53, 55, 56] had been assigned a lower quality judgment. The meta-analyses revealed a small effect *g* = 0.34, *p* = .004, 95% CI [0.11, 0.56], indicating higher levels of multidimensional caregiver sensitivity among secure versus insecure children (See Fig 5). There was a moderate degree of true between study heterogeneity (*Q* = 10.52, *p* = .06, *I*^2^ = 54.30%). The results of Egger’s regression test [51] for funnel plot asymmetry was non-significant (*p* = .48), suggesting no evidence of publication bias.

Five separate moderator analyses were conducted to determine if the longitudinal relationship between multidimensional sensitivity and secure versus insecure attachment varies as a function of key study variables. The moderator analyses were non-significant for quality score (Q_b_ = 2.39, *p* = 0.12), child gender (Q_b_ = .34, *p* = 0.56), sample type (clinical vs. normative; Q_b_ = 0.80, *p* = 0.37), child age at attachment (Q_b_ = 1.56, *p* = 0.22), and socioeconomic status (Q_b_ = 1.31, *p* = 0.25).

*1*.*2*.*2*. *Secure vs*. *insecure*: *Qualitative synthesis*. Four studies were included in the qualitative synthesis examining differences in multidimensional caregiver sensitivity for secure versus insecure children [26, 43, 54, 57]. All of the studies were drawn from the same sample as a study [55] that was prioritized for the quantitative synthesis.

For the present qualitative synthesis, all studies consisted of a normative sample. Two of the studies were judged to have a higher quality [26, 43] and two of the studies were judged to have a lower quality [54, 57]. Although all of the studies consisted of the same sample, variations in the methodological quality judgment was due to variability in reporting the required information to be considered as higher versus lower. Between-group effect sizes were calculated to examine the direction and magnitude of the differences in multidimensional caregiver sensitivity among caregivers of children who are secure versus insecure. There were small to medium overall effects in these studies (Hedges’ *g* ranging from .32 to .49) suggesting that multidimensional caregiver sensitivity was higher for caregivers of children who were secure versus insecure. Variations in effect sizes across studies drawn from the same sample is likely a result of the variation in sample sizes (see Table 1), and the variation in study provided statistical data used to calculate the effect sizes (e.g., ANOVA, correlation, and pooled means and standard deviations).

**Fig 6:**
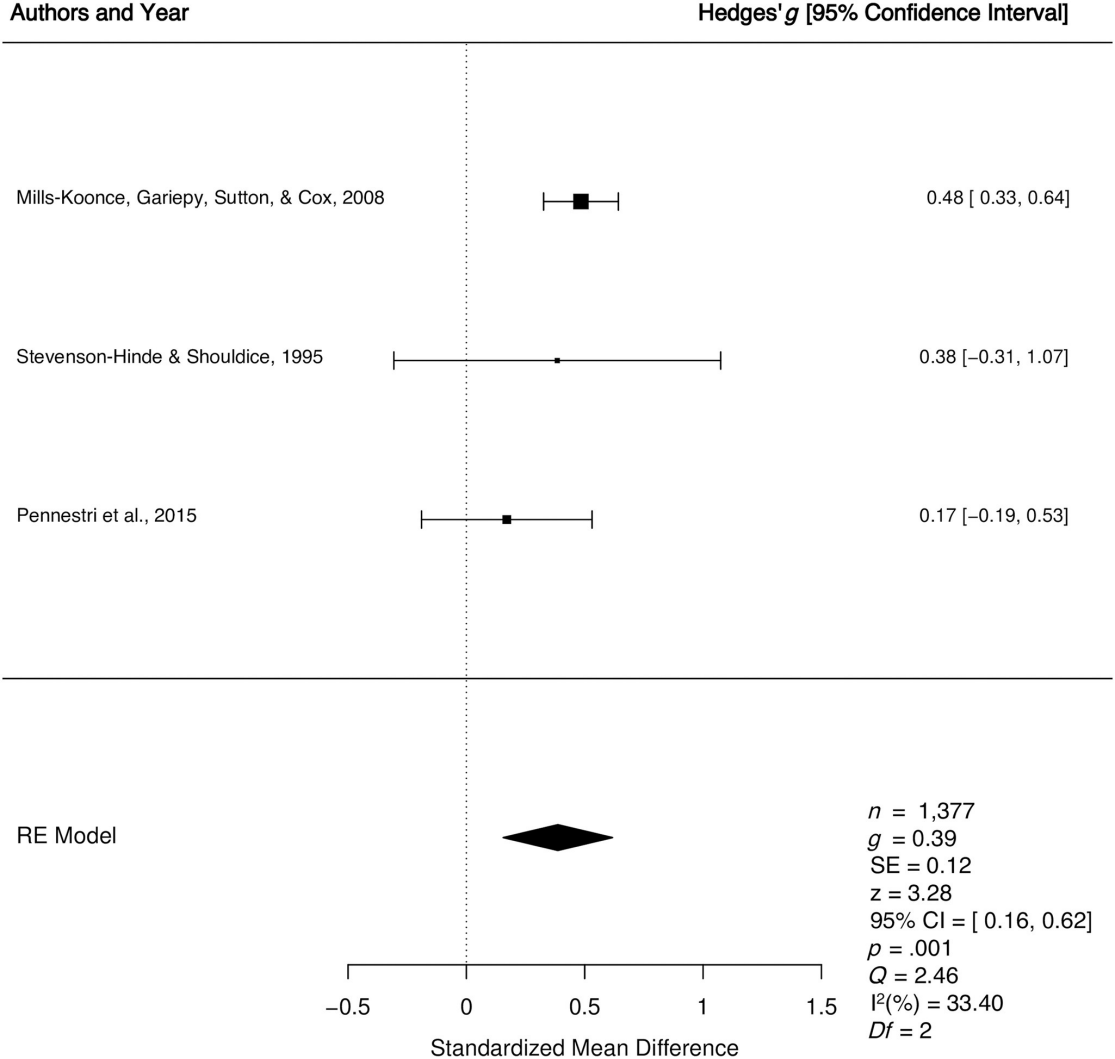
Forest plot for the longitudinal relationship between multidimensional caregiver sensitivity and organized versus disorganized preschool attachment. RE = Random Effects Model; *g* = Hedges’ *g; Q* = Cochran’s heterogeneity statistic; I^2^ = percentage of variability across studies that is due to between-study heterogeneity.

*1*.*2*.*3*. *Organized vs*. *disorganized*: *Quantitative synthesis*. Three studies were included in the quantitative synthesis examining differences in multidimensional caregiver sensitivity for organized versus disorganized children. The weighted mean effect size of differences in multidimensional caregiver sensitivity for children who were organized versus disorganized was calculated from a total sample of 1, 377 child-caregiver dyads, with one clinical sample [56] and two normative samples [50, 55]. All three studies [50, 55, 56] had been assigned a lower quality judgement. The meta-analyses revealed a small effect *g* = 0.39, *p* = .001, 95% CI [0.16, 0.62], indicating higher levels of multidimensional caregiver sensitivity among caregivers of organized versus disorganized children (See Fig 6). There was a small to moderate degree of true between study heterogeneity (*Q* = 2.46, *p* = .29, *I*^2^ = 33.40%). The results of Egger’s regression test [51] for funnel plot asymmetry was non-significant (*p* = .51), suggesting no evidence of publication bias.

Four separate moderator analyses were conducted to determine if the longitudinal relationship between multidimensional sensitivity and organized versus disorganized attachment varies as a function of key study variables. The moderator analyses were non-significant for quality score (Q_b_ = 2.40, *p* = 0.12), child gender (Q_b_ = 2.45, *p* = 0.12), sample type (clinical vs. normative; Q_b_ = 2.38, *p* = 0.12), and child age that preschool attachment was assessed (Q_b_ = 0.001, *p* = 0.98). Moderator analyses could not be conducted for socioeconomic status (low vs. middle/high) due to lack of variability in the studies (i.e., all samples were identified as having a high/middle socioeconomic status).

*1*.*2*.*4*. *Organized vs*. *disorganized*: *Qualitative synthesis*. Three studies were included in the qualitative synthesis examining differences in multidimensional caregiver sensitivity for organized versus disorganized children [26, 57, 58]. The studies [26, 57, 58] were drawn from the same samples of articles [55, 56] that were prioritized for the quantitative synthesis.

For the present qualitative synthesis, one study [58] consisted of a clinical sample, and two studies [26, 57] consisted of a normative sample. Two of the studies were judged to have a higher quality [26, 58] and one of the studies was judged to have a lower quality [57]. Between-group effect sizes were calculated to examine the direction and magnitude of the differences in multidimensional caregiver sensitivity among caregivers of children who are organized versus disorganized. Among the two studies [26, 57] drawn from the same sample, one study [57] had a small overall effect (*g* = .30) and the other [26] had medium overall effect (*g* = .47), suggesting that multidimensional caregiver sensitivity was higher for caregivers of children who were organized versus disorganized. Variations in effect sizes across the same sample is likely a result of the variation in sample sizes (see Table 1). Another study [58] had a medium (approaching large) overall effect (*g* = .61), again supporting the finding that caregiver sensitivity is higher for caregivers of children who are organized relative to disorganized. Of note, the study statistics used to calculate the aforementioned effect size [58], implemented several control variables in the analysis (i.e., child birthweight, child genetic markers, child gender, maternal mental health, maternal demographic variables) and examined caregiver sensitivity as a predictor of organization on a rating scale rather than implementing the organized/disorganized dichotomy.

### 2. Concurrent relationship between caregiver sensitivity and preschool attachment

#### 2.1. Concurrent associations between unidimensional caregiver sensitivity and preschool attachment

Seventeen studies examined the concurrent relationship between unidimensional caregiver sensitivity and preschool attachment [16, 17, 32, 44, 47–50, 59–67].

**Fig 7:**
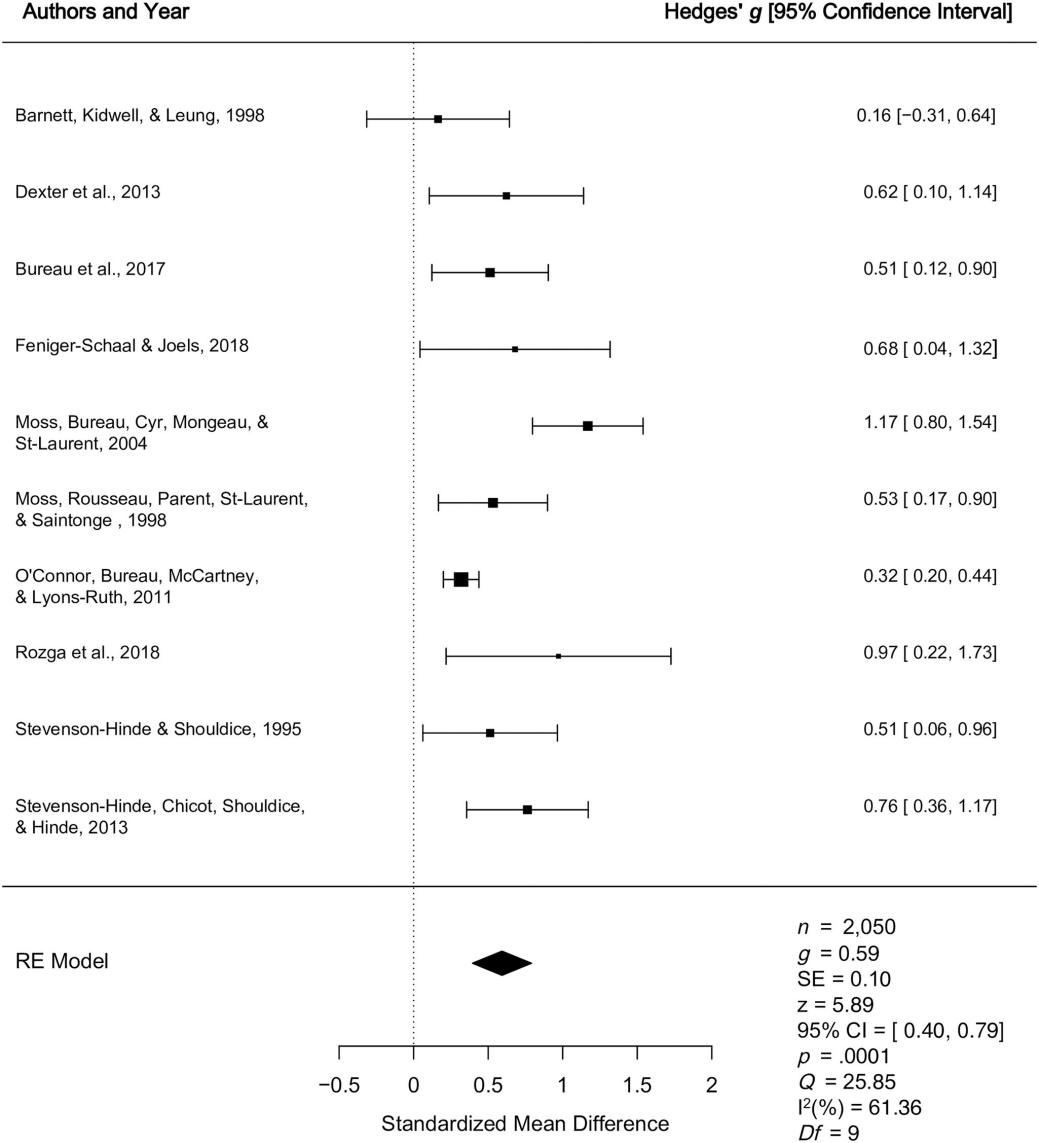
Forest plot for the concurrent relationship between unidimensional caregiver sensitivity and secure versus insecure preschool attachment. RE = Random Effects Model; *g* = Hedges’ *g; Q* = Cochran’s heterogeneity statistic; Q; I^2^ = percentage of variability across studies that is due to between-study heterogeneity.

*2*.*1*.*1*. *Secure vs*. *insecure*: *Quantitative synthesis*. Ten studies were included in the quantitative synthesis examining differences in unidimensional caregiver sensitivity for secure versus insecure children. The weighted mean effect size of differences in unidimensional caregiver sensitivity for children who were secure versus insecure was calculated from a total sample of 2, 050 caregiver-child dyads, that consisted of four clinical samples [44, 59, 60, 66] and six normative samples [16, 50, 62, 63, 65, 67]. Five [16, 44, 59, 62, 65] studies had been assigned a higher quality judgment and five studies [50, 60, 63, 66, 67] had been assigned a lower quality judgment. The meta-analyses revealed a medium effect *g* = 0.59, *p* < .0001, 95% CI [0.40, 0.79], indicating higher levels of unidimensional caregiver sensitivity among caregivers of secure versus insecure children (See Fig 7). There was a moderate degree of true between study heterogeneity (*Q* = 25.85, *p* = .002, *I*^2^ = 61.36%). The results of Egger’s regression test [51] for funnel plot asymmetry was non-significant (*p* = .27), suggesting no evidence of publication bias.

Five separate moderator analyses were conducted to determine if the concurrent relationship between unidimensional sensitivity and secure versus insecure attachment varies as a function of key study variables. The moderator analyses were non-significant for quality score (Q_b_ = 0.03, *p* = 0.86), child gender (Q_b_ = 3.05, *p* = 0.08), sample type (clinical vs. normative; Q_b_ = 0.05, *p* = 0.80), age that preschool attachment was assessed (Q_b_ = 0.01, *p* = 0.91), and socioeconomic status (Q_b_ = 0.36, *p* = 0.16). Of note, the moderator analysis for socioeconomic status included one less study [62], given that socioeconomic status had already been controlled for in the study’s original analysis.

*2*.*1*.*2*. *Secure vs*. *insecure*: *Qualitative synthesis*. Seven studies were included in the qualitative synthesis examining differences in unidimensional caregiver sensitivity for secure versus insecure children [17, 32, 47–49, 61, 64]. Two of the studies [32, 49] did not provide sufficient data to be included in the quantitative synthesis and five studies [17, 47, 48, 61, 64] were drawn from the same samples of articles [16, 62, 63] prioritized for quantitative synthesis.

For the present qualitative synthesis, two studies [32, 49] consisted of a clinical sample, and five studies [17, 47, 48, 61, 64] consisted of a normative sample. Four of the studies were judged to have a higher quality [17, 47, 48, 61] and three of the studies were judged to have a lower quality [32, 49, 64]. Between-group effect sizes were calculated to examine the direction and magnitude of the differences in unidimensional caregiver sensitivity among caregivers of children who are secure versus insecure. Among the studies [47, 48, 61, 64] drawn from two different samples within the same research group, the overall effect ranged from a medium (bordering large) effect (*g* = .61) to a very large effect (*g* = 1.09), suggesting that unidimensional caregiver sensitivity was higher for caregivers of children who were secure versus insecure. This finding was supported by a study [17] from another research group also identifying an overall medium effect (*g* = .49). The two remaining studies had insufficient data to calculate a between-groups effect size. Both of the studies [32, 49] identified a non-significant relationship between caregiver sensitivity and preschool attachment. Of note, one study [32] analyzed the overall relationship between caregiver sensitivity and the four categories of preschool attachment. Interestingly, both studies reporting non-significant findings [32, 49] were from clinical samples, judged to have lower methodological quality, and were identified as having lower socioeconomic status, in comparison to the studies in the qualitative synthesis identified to have a medium to very large overall effect size.

**Fig 8:**
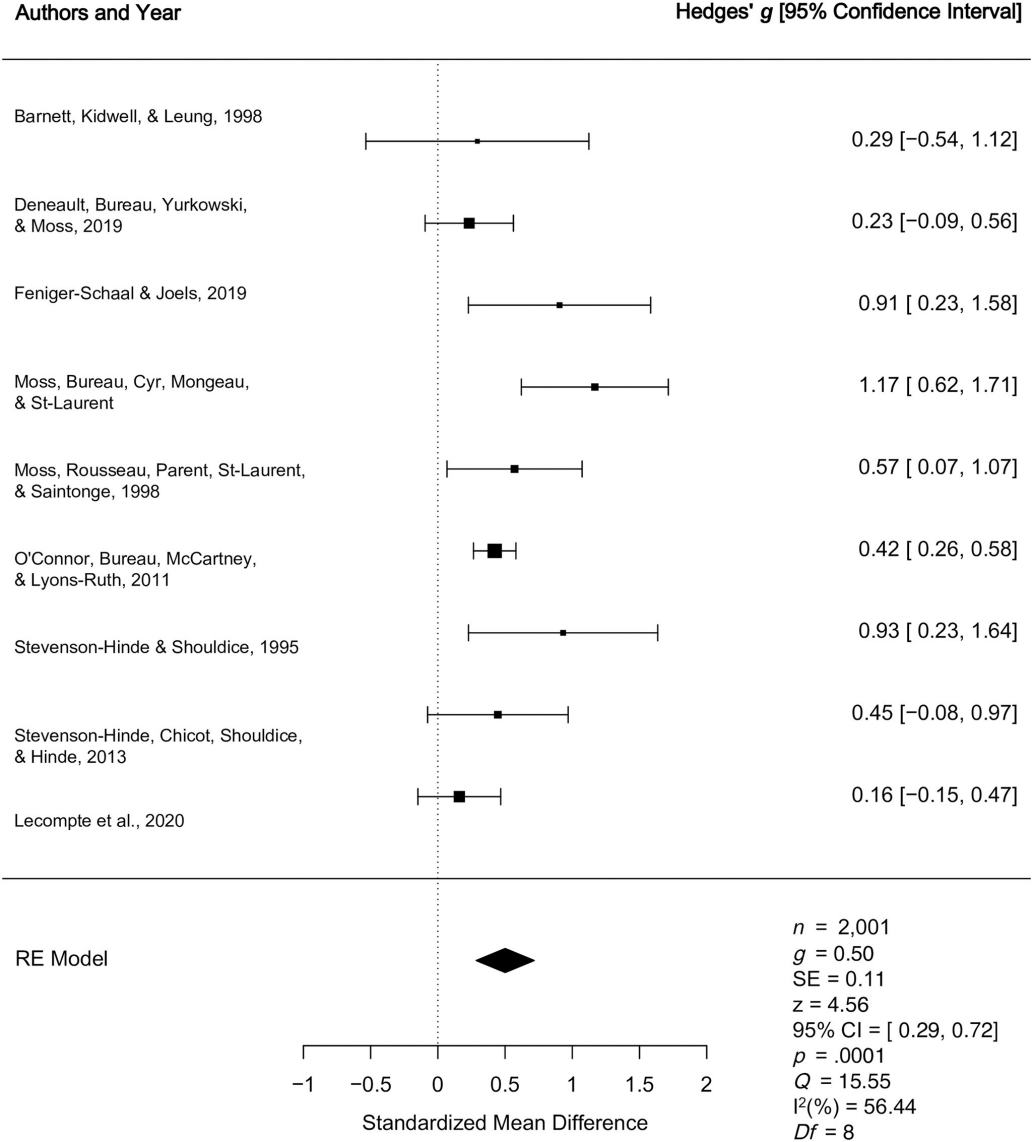
Forest plot for the concurrent relationship between unidimensional caregiver sensitivity and organized versus disorganized preschool attachment. RE = Random Effects Model; *g* = Hedges’ *g; Q* = Cochran’s heterogeneity statistic; Q; I^2^ = percentage of variability across studies that is due to between-study heterogeneity.

*2*.*1*.*3*. *Organized vs*. *disorganized*: *Quantitative synthesis*. *N*ine studies were included in the quantitative synthesis examining differences in unidimensional caregiver sensitivity for organized versus disorganized children. The weighted mean effect size of differences in unidimensional caregiver sensitivity for children who were organized versus disorganized was calculated from a total sample of 2, 001 caregiver-child dyads, that consisted of four clinical samples [17, 44, 59, 68] and five normative samples [50, 62, 63, 65, 67]. Six [17, 44, 59, 62, 65, 68] studies had been assigned a higher quality judgment and three studies [50, 63, 67] had been assigned a lower quality judgment. The meta-analyses revealed a medium effect *g* = 0.50, *p* < .0001, 95% CI [0.29, 0.72], indicating higher levels of unidimensional caregiver sensitivity among caregivers of organized versus disorganized children (See Fig 8). There was a moderate degree of true between study heterogeneity (*Q* = 15.55, *p* = .05, *I*^2^ = 56.44%). The results of Egger’s regression test [51] for funnel plot asymmetry was non-significant (*p* = .10), suggesting no evidence of publication bias.

Five separate moderator analyses were conducted to determine if the concurrent relationship between unidimensional sensitivity and organized versus disorganized attachment varies as a function of key study variables. The moderator analyses were non-significant for child gender (Q_b_ = 2.89, *p* = 0.09), quality score (Q_b_ = 0.42, *p* = 0.52), sample type (clinical vs. normative; Q_b_ = 0.49, *p* = 0.48), age that preschool attachment was assessed (Q_b_ = 0.48, *p* = 0.49), and socioeconomic status (Q_b_ = 0.06, *p* = 0.81). Of note, the moderator analysis for socioeconomic status included one less study [62], given that socioeconomic status had already been controlled in the study’s original analysis.

*2*.*1*.*4*. *Organized vs*. *disorganized*: *Qualitative synthesis*. Six studies were included in the qualitative synthesis examining differences in unidimensional caregiver sensitivity for organized versus disorganized children [32, 47–49, 61, 64]. Two of the studies [32, 49] did not provide sufficient data to be included in the quantitative synthesis and the remaining four studies [47, 48, 61, 64] utilized samples from studies [62, 63] that were already prioritized for the quantitative analysis.

For the present qualitative synthesis, two studies [32, 49] consisted of a clinical sample, and four studies [47, 48, 61, 64] consisted of a normative sample. Three of the studies were judged to have a higher quality [47, 48, 61] and three of the studies were judged to have a lower quality [32, 49, 64]. Between-group effect sizes were calculated to examine the direction and magnitude of the differences in unidimensional caregiver sensitivity among caregivers of children who are organized versus disorganized. Among the studies [47, 48, 61, 64] drawn from two different samples within the same research group, the overall effect ranged from a medium effect (*g* = .42) to a very large effect (*g* = 1.66), suggesting that unidimensional caregiver sensitivity was higher for caregivers of children who were organized versus disorganized. The study [48] with the largest effect size (*g* = 1.66) had a sample that was more than double that of the other studies from the same research group [47, 61, 64] owing to combining participants from two separate cohorts. The two additional studies [32, 49] identified a non-significant relationship between caregiver sensitivity and preschool attachment. Of note, one study [32] analyzed the overall relationship between caregiver sensitivity and the four categories of preschool attachment. Interestingly, both studies reporting non-significant findings were from clinical samples, judged to have lower methodological quality, and were identified as having lower socioeconomic status, in comparison to the studies in the qualitative synthesis identified to have a medium to very large overall effect size.

#### 2.2. Concurrent associations between multidimensional caregiver sensitivity and preschool attachment

Twelve studies examined the concurrent relationship between multidimensional caregiver sensitivity and preschool attachment [15, 43, 45, 53, 55, 67, 69–74].

**Fig 9:**
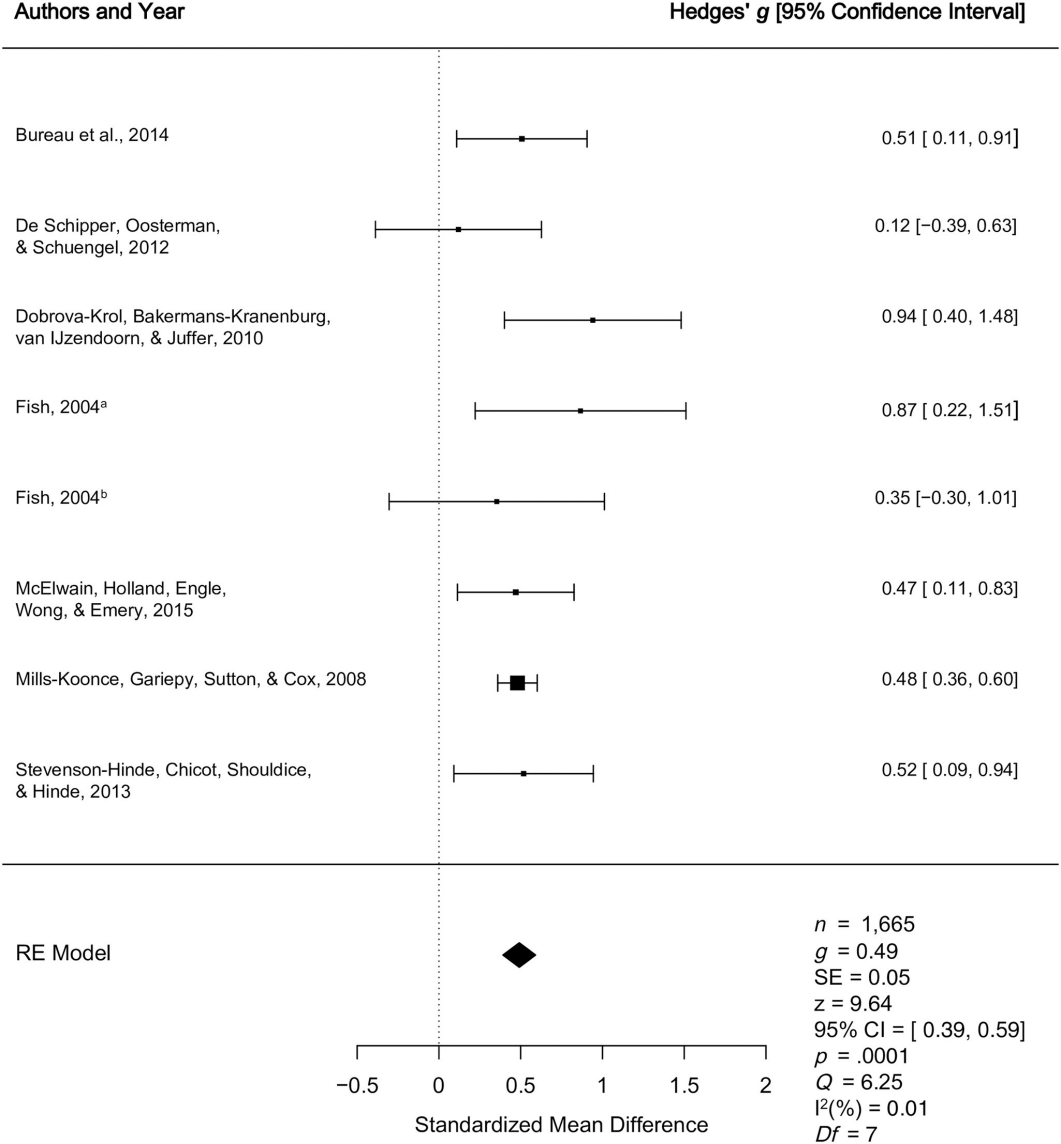
Forest plot for the concurrent relationship between multidimensional caregiver sensitivity and secure versus insecure preschool attachment. RE = Random Effects Model; *g* = Hedges’ *g; Q* = Cochran’s heterogeneity statistic; Q; I^2^ = percentage of variability across studies that is due to between-study heterogeneity.

*2*.*2*.*1*. *Secure vs*. *insecure*: *Quantitative synthesis*. *A* total of seven studies were included in the quantitative synthesis examining differences in multidimensional caregiver sensitivity for secure versus insecure children. It is important to note that among the seven studies, one study was treated as two separate studies and entered twice [53] because separate analyses were run for children with a secure versus insecure infant attachment history and the necessary statistical information to combine these effects to enter it as one study was not available. The weighted mean effect size of differences in multidimensional caregiver sensitivity for children who were secure versus insecure was calculated from a total sample of 1, 665 caregiver-child dyads, that consisted of three clinical samples [53, 69, 70] and four normative samples [15, 55, 67, 72]. Three [15, 70, 72] studies had been assigned a higher quality judgment and four studies [53, 55, 67, 69] had been assigned a lower quality judgment. The meta-analyses revealed a medium effect *g* = 0.49, *p* < .0001, 95% CI [0.39, 0.59], indicating higher levels of multidimensional caregiver sensitivity among secure versus insecure children (See Fig 9). The test of heterogeneity revealed that almost none of the heterogeneity is due to true between-study heterogeneity (*Q* = 6.25, *p* = .51, *I*^2^ = 0.01%). The results of Egger’s regression test [51] for funnel plot asymmetry was non-significant (*p* = .62), suggesting no evidence of publication bias.

Five separate moderator analyses were conducted to determine if the concurrent relationship between multidimensional sensitivity and organized versus disorganized attachment varies as a function of key study variables. The moderator analyses were non-significant for quality score (Q_b_ = 0.16, *p* = 0.69), child gender (Q_b_ = 1.31, *p* = 0.25), sample type (clinical vs. normative; Q_b_ = 0.17, *p* = 0.68), age that preschool attachment was assessed (Q_b_ = 0.01, *p* = 0.93), and socioeconomic status (Q_b_ = 0.17, *p* = 0.68). Of note, the moderator analysis for mean years of age at preschool attachment assessment included one less study [15] given that this variable had already been controlled for in the study’s original analysis.

*2*.*2*.*2*. *Secure vs*. *insecure*: *Qualitative synthesis*. Five studies were included in the qualitative synthesis examining differences in multidimensional caregiver sensitivity for secure versus insecure children [43, 45, 71, 73, 74]. One of the studies [73] did not provide sufficient data to be included in the quantitative synthesis and four studies [43, 45, 71, 74] were drawn from the same samples of papers [55, 72] prioritized for the quantitative synthesis.

For the present qualitative synthesis, one study [73] consisted of a clinical sample, and four studies [43, 45, 71, 74] consisted of a normative sample. One of the studies was judged to have a higher quality [43] and four of the studies were judged to have a lower quality [45, 71, 73, 74]. Between-group effect sizes were calculated to examine the direction and magnitude of the differences in multidimensional caregiver sensitivity among caregivers of children who are secure versus insecure. One of the studies [43] had an overall small effect (*g* = .35), two of the studies [45, 71] had medium effects (*g* = .45 and .41, respectively), and one of the studies [74] had an overall large effect (*g* = .74), indicating that multidimensional caregiver sensitivity is higher among caregivers with secure children relative to insecure children. The remaining study [73] with insufficient data to calculate a between-groups effect size reported a non-significant relationship between a multidimensional measure of caregiver sensitivity and secure versus insecure preschool attachment.

**Fig 10:**
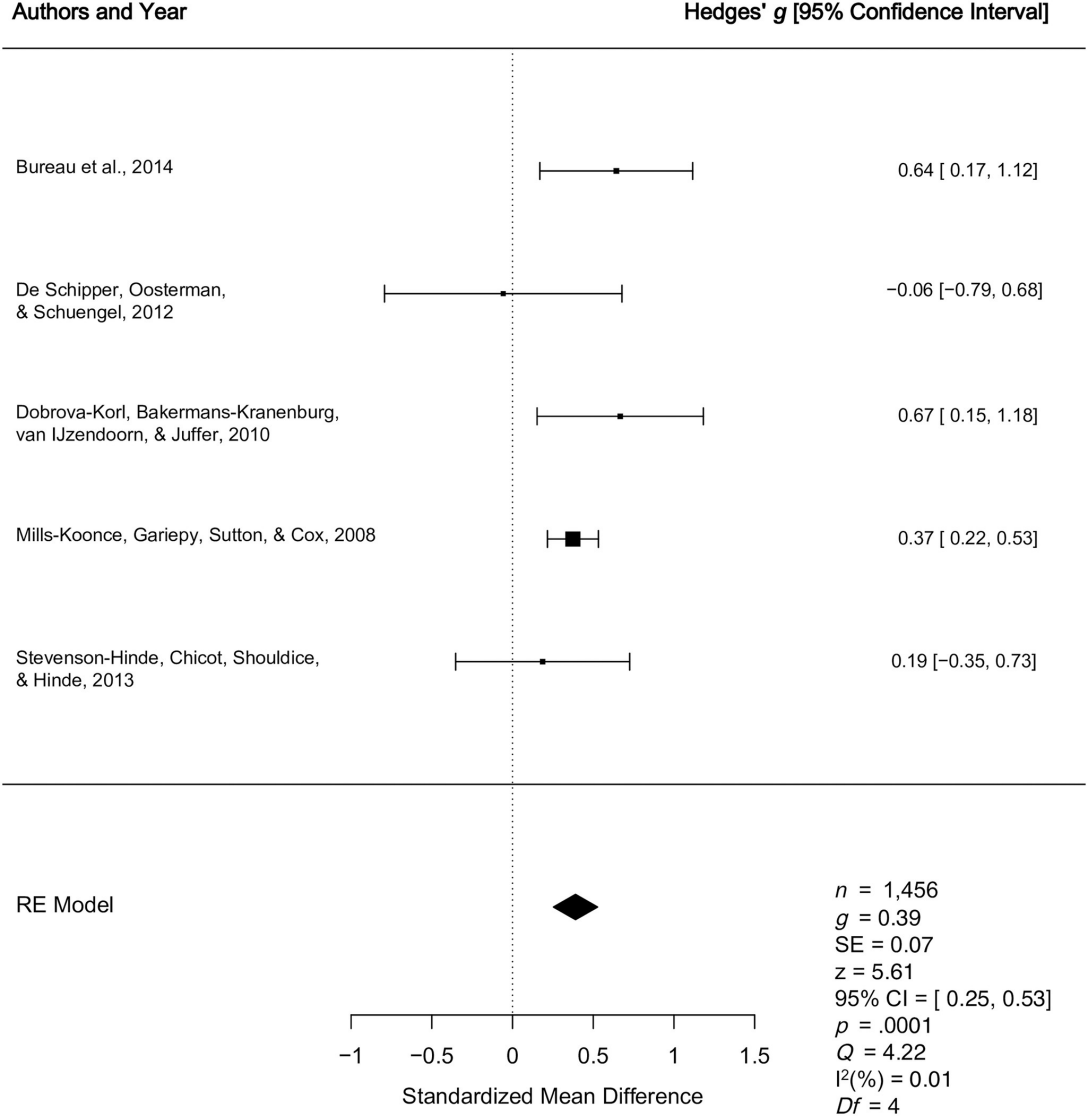
Forest plot for the concurrent relationship between multidimensional caregiver sensitivity and organized versus disorganized preschool attachment. RE = Random Effects Model; *g* = Hedges’ *g; Q* = Cochran’s heterogeneity statistic; Q; I^2^ = percentage of variability across studies that is due to between-study heterogeneity.

*2*.*2*.*3*. *Organized vs*. *disorganized*: *Quantitative synthesis*. Five studies were included in the quantitative synthesis examining differences in multidimensional caregiver sensitivity for organized versus disorganized children. The weighted mean effect size of differences in multidimensional caregiver sensitivity for children who were organized versus disorganized was calculated from a total sample of 1, 465 caregiver-child dyads, that consisted of two clinical samples [69, 70] and three normative samples [15, 55, 67]. Two [15, 70] studies had been assigned a higher quality judgment and three studies [55, 67, 69] had been assigned a lower quality judgment. The meta-analyses revealed a small effect *g* = 0.39, *p* < .0001, 95% CI [0.25, 0.53], indicating higher levels of multidimensional caregiver sensitivity among organized versus disorganized children (See Fig 10). The test of heterogeneity revealed that almost none of the heterogeneity is due to true between-study heterogeneity (*Q* = 4.22, *p* = .37, *I*^2^ = 0.01%). The results of Egger’s regression test [51] for funnel plot asymmetry was non-significant (*p* = .76), suggesting no evidence of publication bias.

Five separate moderator analyses were conducted to determine if the concurrent relationship between multidimensional sensitivity and organized versus disorganized attachment varies as a function of key study variables. The moderator analyses were non-significant for quality score (Q_b_ = 0.00, *p* = 0.24), child gender (Q_b_ = 0.06, *p* = 0.80), sample type (clinical vs. normative; Q_b_ = 0.04, *p* = 0.85), age that preschool attachment was assessed (Q_b_ = 0.21, *p* = 0.65), and socioeconomic status (Q_b_ = 0.04, *p* = 0.85). Of note, the moderator analyses for mean years of age at preschool attachment assessment included one less study [15] given that this variable had already been controlled for in the study’s original analysis.

*2*.*2*.*4*. *Organized vs*. *disorganized*: *Qualitative synthesis*. *O*ne study was included in the qualitative synthesis examining differences in multidimensional caregiver sensitivity for organized versus disorganized children [73]. The study consisted of a clinical sample, and was judged to have lower quality. Insufficient data was reported in order to calculate a between-groups effect size. The study reported a non-significant relationship between a multidimensional measure of caregiver sensitivity and preschool attachment. Of note, the study only tested the overall relationship between caregiver sensitivity and the four attachment categories.

## Discussion

Although past reviews have examined the longitudinal and/or the concurrent relationship between caregiver sensitivity and child attachment outcomes [7, 21–25, 75), this is the first study to systematically review and meta-analyze the relationship between caregiver sensitivity and preschool attachment measured specifically by the Cassidy and Marvin [13] and Main and Cassidy [12] coding systems. Overall, the results of the present review demonstrate that caregiver sensitivity is associated with preschool attachment, both longitudinally and concurrently. Furthermore, regardless of whether caregiver sensitivity is implemented as a unidimensional or multidimensional measure, the quantitative and qualitative syntheses consistently demonstrated that higher levels of caregiver sensitivity is related to a greater likelihood of secure and organized preschool attachment compared to insecure and disorganized preschool attachment, respectively.

The meta-analytic findings of the present review are actually quite congruent with older reviews examining the relationship between caregiver sensitivity and preschool attachment. While previous meta-analytic studies most often reported effect sizes in terms of correlational values, the present meta-analyses presented results in terms of Hedges’ *g*. Given a much higher value of Hedges’ *g* is required to be congruent with a lesser correlational value (i.e., *r* = .*20 or d* = .40) the findings were in line with past work. Second, a recent publication [42] asserts that Cohen’s [40] traditional categorizations for effect sizes are too stringent and offers new interpretations for effect sizes. Also, differentiating from past work, the present study interpreted the findings in accordance with more current guidelines. The eight primary meta-analyses in the present review yielded small to medium effect sizes, similar to past reviews, examining the relationship between caregiver sensitivity to attachment in infancy [7, 21, 25]. Moreover, consistent with a previous review completed by Atkinson and colleagues [21], the present review generally demonstrated stronger effect sizes for studies examining the concurrent relationships relative to longitudinal relationships. In instances where the effect sizes differed from that of past reviews [24, 75], the variability in findings can be explained through a closer examination of the details. For example, in Lucassen and colleague’s [75] review, a small effect was identified for the relationship between caregiver sensitivity and infant attachment. However, attachment in the review was specific to *infant*’s attachment to fathers. Moreover, in Koehn and Kerns’ [24] review, the meta-analytic values were both smaller and larger than the effect sizes denoted in the present study, but this review did not focus on a specific age group (i.e., early childhood to adulthood) and it examined the relationship between caregiver responsiveness and each of the four main attachment categories (i.e. secure, avoidant, ambivalent, disorganized). In order to further explore the present review findings, the subsequent section will discuss the longitudinal synthesis followed by the concurrent synthesis.

### The longitudinal relationship between caregiver sensitivity and preschool attachment

One of the primary goals of the present study was to examine both the longitudinal and concurrent relationship between caregiver sensitivity and preschool attachment. In terms of longitudinal attachment, the results of the meta-analyses demonstrated a small to medium effect, with higher levels of caregiver sensitivity predicting greater secure and organized attachment in preschool, relative to insecure and disorganized attachment styles. Additionally, although most of the moderator analyses were non-significant, the longitudinal relationship between unidimensional caregiver sensitivity and secure versus insecure attachment had larger differences for studies with children who were older when they completed the attachment assessment. Thus, the longitudinal association between earlier unidimensional caregiver sensitivity predicting preschool attachment was stronger when preschoolers were older versus younger. This finding is parallel to the literature reviewing the relationship between caregiver sensitivity and attachment measured by the infant system which reported that there were stronger effect sizes when infants were older when they completed the attachment procedure [7]. Although one could interpret the present and past findings to indicate that a bigger time gap between assessments of caregiver sensitivity and infant or preschool attachment leads to better concordance, this interpretation contradicts other related findings. DeWolff and van IJzendoorn [7] reported that a shorter time interval between caregiver sensitivity and infant attachment assessments led to greater effect sizes. The notion of stronger effect sizes with smaller time gaps is also observed in the present review whereby effect sizes were relatively larger for the concurrent meta-analyses compared to the longitudinal meta-analyses. An alternative and more likely interpretation of the findings that effect sizes are larger when attachment is assessed at a greater age may indicate that attachment assessed later in an infant’s or child’s life is more reliable. Another consideration for the moderator effect of the age at assessment of attachment is in regards to the developmental trajectory between the child’s age at assessment of caregiver sensitivity and the child’s age at assessment of attachment. While only based on three studies, it is noteworthy that while the effect sizes included in this meta-analysis increased as the age at assessment of attachment increased, they also increased with an increase in the child age at which caregiver sensitivity was assessed- with the lowest effect size occurring when caregiver sensitivity occurred at 8 months [46] and the largest occurring when caregiver sensitivity was assessed when the child was 4 years of age [48]. Developmentally, it is important to highlight that in early infancy (8 months) the caregiver and infant are still in the early stages of their relationship, whereas in preschool (4 years of age) caregiver responses to their child are more established thereby explaining the stronger longitudinal relationship between caregiver sensitivity and preschool attachment when caregiver sensitivity was assessed when children were older.

Our analyses suggest the intricacies of the longitudinal relationship are better understood by examining how the relationship differs according to the measurement of caregiver sensitivity. In instances where a unidimensional measure of caregiver sensitivity was employed, the effect size was relatively larger (medium effect) compared to when a multidimensional measure (small effect) of caregiver sensitivity was employed. The differences in the longitudinal relationship when caregiver sensitivity was operationalized as a unidimensional versus a multidimensional measure may be explained by several factors. First, in terms of the grouping of articles, it is more likely that the unidimensional measures were more similar than the multidimensional measures. Unidimensional measures of caregiver sensitivity were operationalized by studies that assessed one single aspect of caregiver behaviour (i.e., a single rating on a sensitivity scale). In contrast, multidimensional measures of caregiver sensitivity were operationalized by studies that combined multiple aspects of caregiver behaviour (i.e., sensitivity, nonintrusiveness, warmth, etc.), but the combination varied pending on the study, thereby creating much more variability among studies identified as employing a multidimensional measure. A second factor to consider is more theoretical in nature. Ainsworth et al.’s [18] original works employed a unidimensional measure of caregiver sensitivity assessed by a single global scale [20]. It was not until subsequent works that other different related behaviours (e.g., warmth, positive affect) were introduced in order to more broadly assess caregiver sensitivity [20]. Accordingly, perhaps introducing new aspects of caregiver behaviour that are related but not the same as sensitivity, results in a weaker longitudinal relationship between caregiver sensitivity and attachment.

The longitudinal relationship between caregiver sensitivity and preschool attachment can be further elucidated through examining the relationship for secure-insecure attachments versus organized-disorganized attachments. Generally, the longitudinal effect sizes were consistently medium when caregiver sensitivity was operationalized as a unidimensional measure and consistently small when caregiver sensitivity was operationalized as a multidimensional measure, regardless of preschool attachment outcomes (i.e., secure vs. insecure, organized vs. disorganized). However, it is noteworthy that in both instances when preschool attachment differences were examined according to organizational status they were slightly larger relative to secure status. This difference is particularly interesting because the syntheses with an organized-disorganized outcome consistently contained fewer studies and a smaller sample size than the studies with a secure-insecure outcome. This finding may suggest that early levels of caregiver sensitivity have a greater impact on determining whether a preschooler is observed to have an organized versus disorganized attachment, relative to a secure versus insecure attachment. This is contrary to expectations because disorganization is conceptualized as associated with frightening or frightened caregiver behaviours, among other factors (i.e., socioeconomic status, caregiver trauma or loss, high stress [76], rather than insensitivity. One important consideration which may help to contextualize these findings is that for the purpose of the meta-analyses, disorganization is merged with role-reversal (i.e., controlling-punitive and controlling-caregiving behaviours), which is not assessed in infancy. Therefore, the concept of organization versus disorganization is more complex in the preschool years, as some may view role-reversal as a form of organization. This may partially explain the results revealed in the present study, but future reviews that separate findings for disorganized versus controlling preschoolers are necessary to shed light on this issue.

### The concurrent relationship between caregiver sensitivity and preschool attachment

Overall, the results of the present review revealed that relative to the longitudinal association, the concurrent relationship between caregiver sensitivity and preschool attachment was slightly stronger. This finding should be considered in the context of the concurrent syntheses consistently including a greater number of studies and a larger sample size relative to the longitudinal syntheses. However, this finding was also identified in a meta-analytic review of caregiver sensitivity and attachment during infancy through toddlerhood [21]. Conceptually, the minor difference in the strength of the concurrent versus longitudinal associations is not surprising due to the closer proximity of time between concurrent assessments of caregiver sensitivity and preschool attachment relative to longitudinal assessments. It is also important to explore the inherent cohesiveness of concurrent assessments relative to longitudinal. In order for studies to be included in the concurrent synthesis it was required that caregiver sensitivity and attachment were assessed within a month of one another. In contrast, while attempts were made to synthesize longitudinal studies as similarly as possible, there was definite variability in the proximity of assessment of caregiver sensitivity and attachment. For example, Pennestri et al. [56] assessed sensitivity at 6 months and preschool attachment at 36 months, whereas studies completed by the NICHD SECCYD [26, 43, 54, 57] often averaged sensitivity at 6, 15, 24, and 36 months.

Despite the above noted differences between the concurrent and longitudinal relationships synthesized, the concurrent synthesis mostly paralleled that of the longitudinal synthesis with regards to the subcategorization operationalizations of caregiver sensitivity (i.e., unidimensional versus multidimensional). As with longitudinal associations, the concurrent relationship between unidimensional measures of caregiver sensitivity and preschool attachment was greater than that of the concurrent relationship between multidimensional caregiver sensitivity and preschool attachment. Similar to the longitudinal associations, it is possible that different findings when caregiver sensitivity was operationalized as a unidimensional measure versus a multidimensional measure, are likely attributed to the greater variability in the studies employing a multidimensional measure, and also potentially explained by deviations from a “pure” assessment of caregiver sensitivity.

In contrast to the longitudinal synthesis, the concurrent synthesis demonstrated that relative to organized-disorganized attachment outcomes, the relationship between caregiver sensitivity and preschool attachment was greater when attachment differences were examined in terms of the secure-insecure dichotomy. One potential explanation for this finding is that when caregiver sensitivity is measured in close proximity to preschool attachment, there are clear differences in the sensitivity of caregivers with secure children relative to insecure children. However, when caregiver sensitivity is measured in further proximity from preschool attachment, changes have occurred in the quality of the sensitivity of caregivers and preschoolers’ attachment status has changed relative to what it may have been when sensitivity was assessed at an earlier date (e.g., children switched from secure to insecure or vice versa). In contrast, possibly caregivers who demonstrate lower sensitivity at infancy are more likely to have lower sensitivity when their children are in preschool, thereby facilitating a consistency in disorganized attachment from infancy to preschool.

### Limitations

There are some limitations that warrant consideration for the present review. Despite our comprehensive search strategy, studies were excluded if they were not in English or French. Another consideration is that although efforts were made to group uniform studies for each of the syntheses conducted, there was inherent variability in some of the studies that were synthesized. For example, given the vast range operationalizations of caregiver sensitivity [20], articles were categorized according to whether they employed a unidimensional measure of caregiver sensitivity (akin to Ainsworth et al.’s [18] original sensitivity construct), or a multidimensional measure of caregiver sensitivity. In addition to the inadvertent variability that this creates, particularly in the multidimensional synthesis, it is important to note that this is only one way to operationalize caregiver sensitivity. Perhaps a different approach to synthesis (e.g., grouping unidimensional and multidimensional measures together) would yield different results. It is also important to consider that the present review only included objective behaviourally coded measures of caregiver sensitivity. Taking a cohesive approach to the present review, studies implementing a measure of caregiver sensitivity through self-completed questionnaires were not included. Moreover, studies that employed a different attachment coding system (e.g., Preschool Assessment of Attachment [PAA]; [31]) than the Cassidy and Marvin [13] and Main and Cassidy [12] systems were not included in the present review to allow for a more focused review. Another consideration of the present review is that studies were only synthesized in terms of differences in caregiver sensitivity according to secure-insecure and organized-disorganized dichotomies due to the limited research available and the need to take other important factors under consideration such as longitudinal versus concurrent and measurement variability. With an increase in studies examining caregiver sensitivity and preschool attachment, future reviews should aim to synthesize a more focused review on one or two aspects of the current syntheses.

A final consideration is that it was not possible to calculate the attenuated effect sizes that account for variability in reliability coding across studies [33], due to limitations in the reliability statistics available for many of the reviewed studies. However, the effect sizes calculated in the present review were similar, if not slightly larger, than past reviews of caregiver sensitivity and attachment [21, 25], including those that have previously calculated the attenuated effect sizes [7]. Therefore, we are confident that the effect sizes yielded in the present review are an adequate representation of the relationship between caregiver sensitivity and preschool attachment in the field. Future reviews of this nature may choose to incorporate Hunter and Schmidt’s [33] attenuation corrections for meta-analysis should the required data be available.

## Conclusions

Overall, the present systematic literature review and meta-analysis provides an updated and nuanced synthesis of the literature linking caregiver sensitivity to preschool attachment outcomes. Due to the critical role ascribed to the first years of life on developmental trajectories [77], these findings are of great import for extending the collective body of literature [7, 21–25] to the preschool age. Identifying caregiver sensitivity as a key factor that has a longitudinal and concurrent impact on preschooler attachment and thus developmental psychopathology, empirically confirms sensitivity as an area for early prevention and intervention. Implementation of programs to assess and improve the sensitivity in which caregivers interact with their infants and young children is paramount to improving attachment and thus mental health outcomes in childhood through adulthood. Further research is necessary in order to understand how caregiver sensitivity may interact with key predictors of child attachment. Additionally, as shown in the present review, more research is required in order to better elucidate the longitudinal relationship between early caregiver sensitivity and preschool attachment.

Moving forward, it will also be imperative for understanding how caregiver sensitivity is related to preschool attachment when caregiver sensitivity is assessed in different and naturalistic contexts. The attachment system is activated in distress, but laboratory distress paradigms are necessarily low to moderately low distress paradigms (free-play, semi-structed play paradigm, mild frustration). Identifying naturally-occurring high distress paradigms for future studies, will almost certainly augment current understanding in the mechanisms subsuming the interrelationships with the distress context, attachment and caregiver sensitivity.

## Supporting information

S1 Appendix PsycINFO search strategy. (PDF)

S2 Appendix PRISMA checklist. (PDF)

S3 Appendix Protocol for ambiguous abstracts. (PDF)

S4 Appendix Quality assessment checklist. (PDF)

S5 Appendix Meta-analysis dataset. (XLSX)

## References

[1] World Health Organization—Child and Adolescent Mental Health [Internet]. Geneva: World Health Organization; c2019 [cited 2019 July 4]. https://www.who.int/mental_health/maternal-child/child_adolescent/en/.

[2] DeKlyen M, Greenberg MT. Attachment and psychopathology in childhood In: Cassidy J, Shaver PR, editors. Handbook of attachment: theory, research, and clinical applications. 3rd ed New York (NY): The Guildford Press; 2016 p. 639–66.

[3] Groh AM, Roisman GI, van IJzendoorn MH, Bakermans-Kranenburg MJ, Fearon RP. The significance of insecure and disorganized attachment for children’s internalizing symptoms: a meta-analytic study. Child Dev. 2012;83(2):591–610. 10.1111/j.1467-8624.2011.01711.x 22235928

[4] Thompson RA. The development of the person: social understanding, relationships, self, conscience In Damo W, Lerner RM, editors. Handbook of child psychology: Vol. 3. social, emotional, and personality development. 6th edition Hoboken (NJ): Wiley; 2006 pp. 24–98

[5] Wright B, Barry M, Hughes E, Trepel D, Ali S, Allgar V, et al Clinical effectiveness and cost-effectiveness of parenting interventions for children with severe attachment problems: a systematic review and meta-analysis. Health Technol Assess. 2015;19(52). 10.3310/hta19520 26177494 PMC4780895

[6] Wright B, Hackney L, Hughes E, Barry M, Glaser D, Prior V, et al Decreasing rates of disorganised attachment in infants and young children, who are at risk of developing, or who already have disorganised attachment. A systematic review and meta-analysis of early parenting interventions. PloS One. 2017;12(7): e0180858 10.1371/journal.pone.0180858 28708838 PMC5510823

[7] De Wolff MS, van IJzendoorn MH. Sensitivity and attachment: a meta-analyses on parental antecedents of infant attachment. Child Dev. 1997;68(4):571–91. 9306636

[8] Bowlby J. Attachment and loss: volume 2. separation. Revised ed. New York: Basic Books; 1973.

[9] Bretherton I. In pursuit of the internal working model construct and its relevance to attachment relationships In: Grossmans KE, Grossmann K, Waters E, editors. Attachment from infancy to adulthood: the major longitudinal studies. New York: The Guildford Press; 2005.

[10] Ainsworth MDS, Blehar MC, Waters E, Wall S. Patterns of attachment: a psychological study of the strange situation. Hillsdale (NJ): Erlbaum; 1978.

[11] Solomon J, George C. The measurement of attachment security and related constructs in infancy and early childhood In Cassidy J, Shaver PR, editors. Handbook of attachment: theory, research, and clinical applications. 3^rd^ edition New York, NY: The Guildford Press; 2016 pp. 366–96.

[12] Main M, Cassidy J. Categories of response to reunion with the parent at age 6: predictable from infant attachment classifications and stable over a 1-month period. Dev.Psychol. 1988;24: 415–26.

[13] Cassidy J, Marvin RS; MacArthur Working Group on Attachment. Attachment organization in three and four year olds: procedures and coding manual. University of Virginia; 1992.

[14] Badovinac S, Martin J, Guérin-Marion C, O’Neill M, Pillai Riddell R, Bureau JF, et al Associations between mother-preschooler attachment and maternal depression symptoms: a systematic review and meta-analysis. PLOS One. 2018;13(10):1–27. 10.1371/journal.pone.0204374 30278066 PMC6168129

[15] Bureau JF, Yurkowski K, Schmiedel S, Martin J, Moss E, Pallanca D. Making children laugh: parent-child dyadic synchrony and preschool attachment. Infant Ment Health J. 2014;35(5):482–94. 10.1002/imhj.21474 25798498

[16] Bureau JF, Martin J, Yurkowski K, Schmiedel S, Quen J, Moss E, et al Correlates of child-father and child-mother attachment in the preschool years. Attach Hum Dev. 2017;19(2):130–50. 10.1080/14616734.2016.1263350 27899058

[17] Deneault AA, Bureau JF, Yurkowski K, Moss E. Validation of the preschool attachment rating scales with child-mother and child-father dyads. Attach Hum Dev. 2019:1–23. 10.1080/14616734.2019.1589546 30873911

[18] Ainsworth MDS, Bell SM, Stayton DJ. Infant-mother attachment and social development: “socialization” as a product of reciprocal responsiveness to signals In: Richards MPM, editors. The integration of a child into a social world. Cambridge: Cambridge University Press; 1974 p. 99–135.

[19] Bowlby J. Attachment and loss: volume 1. attachment. Revised ed. Harmondsworth: Penguin; 1969.

[20] Mesman J, Emmen RAG. Mary Ainsworth’s legacy: a systematic review of observational instruments measuring parental sensitivity. Attach Hum Dev. 2013;15: 485–506. 10.1080/14616734.2013.820900 24299131

[21] Atkinson L., Niccols A, Paglia A., Coolbear J., Parker K, Poulton L, et al A meta-analysis of time between maternal sensitivity and attachment assessments: Implications for internal working models in infancy/toddlerhood. J Soc Pers Relat. 2000;17(6):791–810. 10.1177/0265407500176005

[22] Deans CL. Maternal sensitivity, it’s relationship with child outcomes, and interventions that address it: a systematic literature review. Early Child Dev Care. 2018 10.1080/03004430.2018.1465415

[23] Goldsmith HH, Alansky JA. Maternal and infant predictors of attachment: a meta-analytic review. J Consult Clin Psychol. 1987;55(6):805–816. 10.1037//0022-006x.55.6.805 3320116

[24] Koehn AJ, Kerns KA. Parent-child attachment: meta-analysis of associations with parenting behaviors in middle childhood and adolescence. Attach Hum Dev. 2018;20(4):378–405. 10.1080/14616734.2017.1408131 29192551

[25] Zeegers MAJ, Colonnesi C, Stams GJJM, Meins E. Mind matters: a meta-analysis on parental mentalization and sensitivity as predictors of infant-parent attachment. Psychol Bull. 2017;143(12):1245–72. 10.1037/bul0000114 28805399

[26] National Institute of Child Health and Human Development. Child care and family predictors of preschool attachment and stability from infancy. Dev Psychol. 2001;37(6): 847–62. 10.1037/0012-1649.37.6.847 11699758

[27] Moher D, Liberati A, Tetzlaff J, Altman D. G. Preferred reporting items for systematic reviews and meta-analyses: the PRISMA statement. Ann Intern Med. 2009;151: 264–9. 10.7326/0003-4819-151-4-200908180-00135 19622511

[28] O’Neill M, Badovinac S, Martin J, Deneault AA, Guérin MC, Goldreich R, et al. Preschool attachment classification system (PACS): a systematic review of maternal and child antecedents, correlates, and consequences of preschool attachment (CRD42017073417). In: PROSPERO: International prospective register of systematic reviews. [Internet]. 2017. https://www.crd.york.ac.uk/prospero/display_record.php?RecordID=73417

[29] Greenberg MT, Speltz ML, Deklyen M, Endriga MC. Attachment security in preschoolers with and without externalizing behaviour problems: a replication. Dev Psychopathol. 1991;3(4):413–30. 10.1017/S0954579400007604

[30] Bretherton I, Ridgeway D, Cassidy J. Assessing internal working models of the attachment relationship: an attachment story completion task for 3-year-olds In: Greenberg MT, Cicchetti D, Cummings EM, editors. Attachment in the preschool years: theory, research, and intervention. Chicago: University of Chicago Press; 1990 p. 273–308.

[31] Crittenden PM. The preschool assessment of attachment. coding manual. 1992. Available from the author.

[32] Crittenden PM, Claussen AH, Kozlowska K. Choosing a valid assessment of attachment for clinical use: a comparative study. Aust N Z J Fam Ther. 2007;28(2):78–87.

[33] Hunter JE, Schmidt FL. Methods of meta-analysis: Correcting error and bias in research findings. 3rd ed Thousand Oaks (CA): Sage.

[34] Sanderson S, Tatt ID, Higgins JP. Tools for assessing quality and susceptibility to bias in observational studies in epidemiology: a systematic review and annotated bibliography. Int J Epidemiol Commun H. 2007;36(3): 666–76. 10.1093/ije/dym018 17470488

[35] National Heart, Lungs, and Blood Institute [Internet]. Bethesda (MD): Quality assessment tool for observational cohort and cross sectional studies- [cited 2017] https://www.nhlbi.nih.gov/health-topics/study-quality-assessment-tools

[36] Downs SH, Black N. The feasibility of creating a checklist for the assessment of the methodological quality both of randomised and non-randomised studies of health care interventions. J Epidemiol Commun H. 1998; 52(6) 377–84. 10.1136/jech.52.6.377 9764259 PMC1756728

[37] Crombie IK. The pocket guide to critical appraisal: a handbook for health care professionals. Hoboken(NJ): Blackwell Publishing; 2007.

[38] Del Re AC. A practical tutorial on conducting meta-analysis in R. Quant Methods Psychol. 2015;11(1):37–50. 10.20982/tqmp/11.1.p037

[39] Viectbauer W. Conducting meta-analyses in R with the metaphor package. J Stat Softw. 2010;36(3): 1–48.

[40] Cohen J. Statistical power analysis for the behavioural science*s*. Revised ed. New York (NY): Academic Press; 1977.

[41] Cohen J. Statistical power analysis for the behavioral sciences. 2nd ed Hillsdale (NJ): Erlbaum; 1988.

[42] Funder DC, Ozer DJ. Evaluating effect size in psychological research: sense and nonsense. Adv Methods Pract Psychol Sci. 2019;2(2):156–168. 10.1177/2515245919847202

[43] Campbell SB, Brownell CA, Hungerford A, Spieker SJ, Mohan R, Blessing JS. The course of maternal depressive symptoms and maternal sensitivity as predictors of attachment security at 36 months. Dev Psychopath. 2004;16(02). 10.1017/s0954579404044499 15487594

[44] Feniger-Schaal R, Joels T. Attachment quality of children with ID and its link to maternal sensitivity and structuring. Dev Disabil Res Rev. 2018;76:56–64. 10.1016/j.ridd.2018.03.004 29567485

[45] Manning LB. The relation between changes in maternal sensitivity and attachment from infancy to 3 years. J Soc Pers Relat. 2019; 36(6):1731–1746. 10.1177/0265407518771217

[46] Meins E, Bureau JF, Fernyhough C. Mother-child attachment from infancy to the preschool years: predicting security and stability. Child Dev. 2018;89(3): 1022–38. 10.1111/cdev.12778 28294294

[47] Moss E, Cyr C, Bureau JF, Tarabulsy GM, Dubois-Comtois K. Stability of attachment during the preschool period. Dev Psychol. 2005;41(5): 773–83. 10.1037/0012-1649.41.5.773 16173874

[48] Moss E, Cyr C, Dubois-Comtois K. Attachment at early school age and developmental risk: examining family contexts and behavior problems of controlling-caregiving, controlling-punitive, and behaviorally disorganized children. Dev Psychol. 2004;40(4): 519–32. 10.1037/0012-1649.40.4.519 15238040

[49] Seifer R, Lagasse LL, Lester B, Bauer C R, Shankaran S, Bada, et al Attachment status in children prenatally exposed to cocaine and other substances. Child Dev. 2004;75(3): 850–68. 10.1111/j.1467-8624.2004.00710.x 15144490

[50] Stevenson-Hinde J, Shouldice A. Maternal interactions and self-reports related to attachment classifications at 4.5 years. Child Dev. 1995;66(3): 583–96. 10.2307/1131936

[51] Egger M, Smith GD, Schneider M, Minder C. Bias in meta-analysis detected by a simple, graphical test. Br Med J. 1997;315:629–34. 10.1136/bmj.315.7109.629 9310563 PMC2127453

[52] Booth CL, Kelly JF. Child care effects on the development of toddlers with special needs. Child Res Q. 2002;17(2):171–96. 10.1016/s0885-2006(02)00144-8

[53] Fish M. Attachment in infancy and preschool in low socioeconomic status rural Appalachian children: stability and change and relations to preschool and kindergarten competence. Dev Psychopathol. 2004;16(2):293–312. 10.1017/s0954579404044529 15487597

[54] McElwain NL, Cox MJ, Burchinal MR., Macfie J. Differentiating among insecure mother—infant attachment classifications: a focus on child—friend interaction and exploration during solitary play at 36 months. Attach Hum Dev. 2003;5(2): 136–64. 10.1080/1461673031000108513 12791564

[55] Mills-Koonce WR, Gariepy JL, Sutton K, Cox MJ. Changes in maternal sensitivity across the first three years: are mothers from different attachment dyads differentially influenced by depressive symptomatology? Attach Hum Dev. 2008;10(3): 299–317. 10.1080/14616730802113612 18821340

[56] Pennestri M, Gaudreau H, Bouvette-Turcot A, Moss E, Lecompte V, Atkinson L, et al Attachment disorganization among children in neonatal intensive care unit: preliminary results. Early Hum Dev. 2015;91(10): 601–6. 10.1016/j.earlhumdev.2015.07.005 26261864

[57] Spieker S, Crittenden PM. Comparing two attachment classification methods applied to preschool strange situations. Clin Child Psychol Psychiatry. 2010; 15(1):97–120. 10.1177/1359104509345878 19914941 PMC3770309

[58] Wazana A, Moss E, Jolicoeur-Martineau A, Graffi J, Tsabari G, Lecompte V et al The interplay of birth weight, dopamine receptor D4 gene (DRD4), and early maternal care in the prediction of disorganized attachment at 36 months of age. Dev Psychopathol. 2015;27(4pt1): 1145–61. 10.1017/S0954579415000735 26439067 PMC5380440

[59] Barnett D, Kidwell SL, Leung KH. Parenting and preschooler attachment among low-Income urban African American families. Child Dev. 1998;69(6):1657–71. 10.1111/j.1467-8624.1998.tb06183.x 9914645

[60] Dexter CA, Wong K, Stacks AM, Beeghly M, Barnett D. Parenting and attachment among low-income African American and Caucasian preschoolers. J Fam Psychol. 2013;27(4):1368–76. 10.1037/a0033341 23795606

[61] Dubois-Comtois K, Cry C, Moss E. Attachment behavior and mother-child conversations as predictors of attachment representations in middle childhood: a longitudinal study. Attach Hum Dev. 2011;13(4):335–57. 10.1080/14616734.2011.584455 21718222

[62] Moss E, Bureau JF, Cyr C, Mongeau C, St-Laurent D. Correlates of attachment at age 3: construct validity of the preschool attachment classification system. Dev Psychol. 2004;40(3): 323–34. 10.1037/0012-1649.40.3.323 15122960

[63] Moss E, Rousseau D, Parent S, St-Laurent D, Saintonge J. Correlates of attachment at school age: maternal reported stress, mother-child interaction, and behavior problems. Child Dev. 1998;69(5): 1390–405. 10.2307/1132273 9839423

[64] Moss E, St-Laurent D. Attachment at school age and academic performance. Dev Psychol. 2001;37(6): 863–74. 10.1037//0012-1649.37.6.863 11699759

[65] O’Connor E, Bureau JF, McCartney K, Lyons-Ruth K. Risks and outcomes associated with disorganized/controlling patterns of attachment at age three years in the national institute of child health & human development study of early child care and youth development. Infant Ment Health. 2011;32(4): 450–72. 10.1002/imhj.20305 21799549 PMC3143498

[66] Rozga A, Hesse E, Main M, Duschinsky R, Beckwith L, Sigman M. A short-term longitudinal study of correlates and sequelae of attachment security in autism. Attach Hum Dev. 2017;20(2): 160–80. 10.1080/14616734.2017.1383489 28959921 PMC5782853

[67] Stevenson-Hinde J, Chicot R, Shouldice A, Hinde CA. Maternal anxiety, maternal sensitivity, and attachment. Attach Hum Dev. 2013;15(5–6): 618–36. 10.1080/14616734.2013.830387 24299138

[68] Lecompte V, Robins S, King L, Solomonova E, Khan N, Moss E, et al Examining the role of mother-child interactions and DNA methylation of the oxytocin receptor gene in understanding child controlling attachment behaviors. Attach Hum Dev. 2020: 1–19. 10.1080/14616734.2019.1708422 31900042

[69] De Schipper JC, Oosterman M, Schuengel C. Temperament, disordered attachment, and parental sensitivity in foster care: differential findings on attachment security for shy children. Attach Hum Dev. 2012;14(4):349–65. 10.1080/14616734.2012.691651 22697469

[70] Dobrova-Krol NA, Bakermans-Kranenburg MJ, van IJzendoorn MH, Juffer F. The importance of quality of care: effects of perinatal HIV infection and early institutional rearing on preschoolers’ attachment and indiscriminate friendliness. J Child Psycho Psychiatry. 2010;51(12):1368–76. 10.1111/j.1469-7610.2010.02243.x 20456538

[71] McElwain NL, Holland AS, Engle JM, Wong MS. Child anger proneness moderates associations between child-mother attachment security and child behavior with mothers at 33 months. J Fam Psychol. 2012;26(1): 76–86. 10.1037/a0026454 22182337

[72] McElwain NL, Holland AS, Engle JM, Wong MS, Emery HT. Child-mother attachment security and child characteristics as joint contributors to young children’s coping in a challenging situation. Infant Child Dev. 2015;24(4): 414–34. 10.1002/icd.1886

[73] O’Connor TG, Marvin RS, Rutter M, Olrick JT, Britner PA, The English And Romanian Adoptees Study Team. Child–parent attachment following early institutional deprivation. Dev Psychopathol. 2003;15(1): 19–38. 10.1017/s0954579403000026 12848433

[74] McElwain NL, Ravindran N, Emery HT, Swartz R. Theory of mind as a mechanism linking mother-toddler relationship quality and child-friend interaction during the preschool years. Soc Dev. 2019;28:998–1015. 10.1111/sode.12377

[75] Lucassen N, Tharner A, Van IJzendoorn MH, Bakermans-Kranenburg MJ, Volling BL, Verhulst FC, et al The association between paternal sensitivity and infant–father attachment security: a meta-analysis of three decades of research. J Fam Psychol. 2011;25(6): 986–92. 10.1037/a0025855 22004434

[76] Granqvist P, Sroufe LA, Dozier M, Hesse E, Steele M, van Ijzendoorn M, et al Disorganized attachment in infancy: a review of the phenomenon and its implications for clinicians and policy-makers. Attach Hum Dev. 2017;19(6): 534–58. 10.1080/14616734.2017.1354040 28745146 PMC5600694

[77] Centre on the Developing Child at Harvard University. Science to policy and practice: three principles to improve outcomes for children and families [Internet].Cambridge: Centre on the Developing Child at Harvard University; 2017 [cited 2020]. https://46y5eh11fhgw3ve3ytpwxt9r-wpengine.netdna-ssl.com/wp-content/uploads/2017/10/HCDC_3PrinciplesPolicyPractice.pdf

