# Supplementary material for: The longitudinal and concurrent relationship between caregiver sensitivity and preschool attachment: A systematic review and meta-analysis
Source: PLoS One. 2021 Jan 22;16(1):e0245061. doi: 10.1371/journal.pone.0245061 (PMC7822304; doi:10.1371/journal.pone.0245061)
Supplement: S1 Appendix — (PDF) [file pone.0245061.s001.pdf]

S1 Appendix. PsycINFO Search Strategy.

1. attachment behavior/ (18574)
2. attachment theory/ (1626)
3. attachment*.tw. (35573)
4. 1 or 2 or 3 (37081)
5. (separation* adj7 reunion*).mp. (365)
6. strange situation*.mp. (1311)
7. Preschool Attachment Classification System*.mp. (7)
8. PACS.mp. (171)
9. cassidy.af. (11148)
10. MacArthur.af. (15753)
11. Attachment Working Group.af. (4)
12. Organized.mp. (38484)
13. Disorganized.mp. (3493)
14. Disorganization.mp. (4058)
15. Controlling.mp. (58808)
16. Insecur*.mp. (10272)
17. Secure.mp. (13635)
18. Security*.mp. (25513)
19. Avoidant*.mp. (6872)
20. Avoidance.mp. (47428)
21. Ambivalent.mp. (5305)
22. Dependent.mp. (130492)
23. Resistant.mp. (17833)
24. 5 or 6 or 7 or 8 or 9 or 10 or 11 or 12 or 13 or 14 or 15 or 16 or 17 or 18 or 19 or 20 or 21 or 22 or 23 (359189)
25. (infan* or baby* or babies or child* or toddler* or schoolchild* or school child* or school age* or pre-school or preschool* or nursery school* or kindergar* or primary school* or elementary school*).mp. (750640)
26. 4 and 24 and 25 (7916)
27. limit 26 to all journals (5124)
