# Supplementary material for: The longitudinal and concurrent relationship between caregiver sensitivity and preschool attachment: A systematic review and meta-analysis
Source: PLoS One. 2021 Jan 22;16(1):e0245061. doi: 10.1371/journal.pone.0245061 (PMC7822304; doi:10.1371/journal.pone.0245061)
Supplement: S3 Appendix — (PDF) [file pone.0245061.s003.pdf]

S3 Appendix. Protocol for ambiguous abstracts.

Abstracts that did not clearly identify the age at which attachment was measured or the type of measurement used to examine attachment were set aside for full text review if they met one of the following criteria:

1. The abstracts were authored by individuals identified to contribute to the Preschool Attachment Classification System (PACS) Manual.
2. The abstracts were authored by key researchers in the field of child attachment.
3. The abstracts were completed using National Institute of Child Health and Development (NICHD) data.

**Authors to Search for "More Info"**	**Justification**
Cassidy	Authored kindergarten attachment coding guidelines; Authored Preschool Attachment Classification System guidelines
Main	Authored kindergarten attachment coding guidelines; Contributor to Preschool Attachment Classification System guidelines
Marvin	Authored the Preschool Attachment Classification System guidelines
Attachment Working Group	Authored the Preschool Attachment Classification System guidelines
MacArthur	Authored the Preschool Attachment Classification System guidelines
Ainsworth	Contributor to Preschool Attachment Classification System guidelines
Beckwith	Contributor to Preschool Attachment Classification System guidelines
Belsky	Contributor to Preschool Attachment Classification System guidelines
Booth	Contributor to Preschool Attachment Classification System guidelines
Bronson	Contributor to Preschool Attachment Classification System guidelines
Crnic	Contributor to Preschool Attachment Classification System guidelines
Easterbrooks	Contributor to Preschool Attachment Classification System guidelines
Greenberg	Contributor to Preschool Attachment Classification System guidelines
LaGasse	Contributor to Preschool Attachment Classification System guidelines
Ridgeway	Contributor to Preschool Attachment Classification System guidelines
Barnard	Contributor to Preschool Attachment Classification System guidelines
Beeghly	Contributor to Preschool Attachment Classification System guidelines
Blacher	Contributor to Preschool Attachment Classification System guidelines
Bretherton	Contributor to Preschool Attachment Classification System guidelines
Carmichael-Olsen	Contributor to Preschool Attachment Classification System guidelines
Cicchetti	Contributor to Preschool Attachment Classification System guidelines
Cummings	Contributor to Preschool Attachment Classification System guidelines
Gottman	Contributor to Preschool Attachment Classification System guidelines
Harmon	Contributor to Preschool Attachment Classification System guidelines
Morisset	Contributor to Preschool Attachment Classification System guidelines
Slough	Contributor to Preschool Attachment Classification System guidelines
Spieker	Contributor to Preschool Attachment Classification System guidelines
Stevenson-Hinde	Contributor to Preschool Attachment Classification System guidelines
Speltz	Contributor to Preschool Attachment Classification System guidelines

Purcell	Contributor to Preschool Attachment Classification System guidelines
Moss	Key researcher in field of attachment
Lecompte	Key researcher in field of attachment
Bureau	Key researcher in field of attachment
Bernier	Key researcher in field of attachment
Tarabulsy	Key researcher in field of attachment
Moran	Key researcher in field of attachment
Sroufe	Key researcher in field of attachment
Van Ijzendoorn	Key researcher in field of attachment
McElwain	Key researcher in field of attachment
Howes	Key researcher in field of attachment
NICHD, National Institute of Child Health and Development	Key researcher in field of attachment
O'Connor	Key researcher in field of attachment
Lyons-Ruth	Key researcher in field of attachment
Pierrehumbert	Key researcher in field of attachment
Pederson	Key researcher in field of attachment
Bailey	Key researcher in field of attachment
Dubois	Key researcher in field of attachment
Cyr	Key researcher in field of attachment
Humber	Key researcher in field of attachment
McCartney	Key researcher in field of attachment
Rousseau	Key researcher in field of attachment
Parent	Key researcher in field of attachment
St-Laurent	Key researcher in field of attachment
Mongeau	Key researcher in field of attachment
Pascuzzo	Key researcher in field of attachment
Crittenden	Key researcher in field of attachment
