# Supplementary material for: The longitudinal and concurrent relationship between caregiver sensitivity and preschool attachment: A systematic review and meta-analysis
Source: PLoS One. 2021 Jan 22;16(1):e0245061. doi: 10.1371/journal.pone.0245061 (PMC7822304; doi:10.1371/journal.pone.0245061)
Supplement: S4 Appendix — (PDF) [file pone.0245061.s004.pdf]

S4 Appendix. Quality assessment checklist.

1. Was the research question or objective in this paper clearly stated?
2. Was the study population clearly specified and defined?
3. Was the participation rate of eligible persons at least 50%?
4. Were all the subjects selected or recruited from the same or similar populations (including the same time period)?
5. Were inclusion and exclusion criteria for being in the study pre-specified and applied uniformly to all participants?
6. Was a sample size justification, power description, variance accounted for or effect estimates provided for the sensitivity and PACS analyses?*
7. For predictor variables (i.e., caregiver sensitivity variables) that can vary in amount or level, did the study examine different levels of the predictor as related to the outcome (i.e., PACS) (e.g., categories of sensitivity levels, or sensitivity measured as continuous variable)?
8. Were the predictor variables (i.e., caregiver sensitivity variables) clearly defined, valid, reliable, and implemented consistently across all study participants?*
9. Was the predictor variable (i.e., caregiver sensitivity) assessed more than once over time?
10. Were measures of the outcome variable (i.e., PACS) clearly defined, valid, reliable, and implemented consistently across all study participants?*
11. Were the outcome assessors (i.e., PACS coders) blinded to the participants' scores on caregiver sensitivity?*
12. Was loss to follow-up after baseline 20% or less (i.e., was the retention rate greater than or equal to 80%)?*
13. Were key potential confounding variables measured and adjusted statistically for their impact on the outcome (PACS)?*
14. Is the distribution of the overall study population by gender (of the child) described?
15. Are the statistical methods described?
16. Have actual probability values been reported (e.g., 0.035 rather than < 0.05) for the main outcomes except where the probability value is less than 0.001?

***Items were used to determine an overall quality judgment (Higher vs. Lower).**
